# Metal–Organic Frameworks in Agriculture

**DOI:** 10.1021/acsami.2c00615

**Published:** 2022-04-08

**Authors:** Sara Rojas, Antonio Rodríguez-Diéguez, Patricia Horcajada

**Affiliations:** † Biochemistry and Electronics as Sensing Technologies Group, Department of Inorganic Chemistry, University of Granada, Av. Fuentenueva s/n, 18071 Granada, Spain; ‡ Advanced Porous Materials Unit (APMU), IMDEA Energy, Av. Ramón de la Sagra, 3, 28935 Móstoles, Madrid, Spain

**Keywords:** metal−organic frameworks, agrochemicals, controlled release, selective adsorption and degradation, sensing

## Abstract

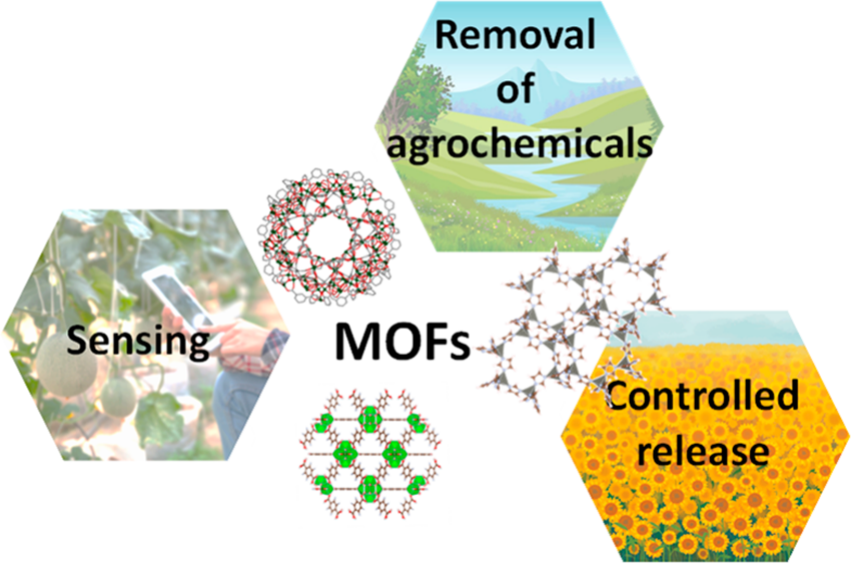

Agrochemicals, which are crucial to meet the world food qualitative and quantitative demand, are compounds used to kill pests (insects, fungi, rodents, or unwanted plants). Regrettably, there are some important issues associated with their widespread and extensive use (e.g., contamination, bioaccumulation, and development of pest resistance); thus, a reduced and more controlled use of agrochemicals and thorough detection in food, water, soil, and fields are necessary. In this regard, the development of new functional materials for the efficient application, detection, and removal of agrochemicals is a priority. Metal–organic frameworks (MOFs) with exceptional sorptive, recognition capabilities, and catalytical properties have very recently shown their potential in agriculture. This Review emphasizes the recent advances in the use of MOFs in agriculture through three main views: environmental remediation, controlled agrochemical release, and detection of agrochemicals.

## 1 Current Challenges of Agriculture

Agrochemicals or agrichemicals (primarily fertilizers and pesticides) have become a fundamental part of today’s agricultural systems in order to fulfill the huge requirement of food. Agrochemicals can be classified on the basis of various principles, such as toxicity, target, chemical composition and formula, mode of entry or action, and source. Here, we will classify them according to their mode of action, although their toxicity, target, and origin will also be discussed. Thus, agrochemicals can be divided into pesticides (insecticides, herbicides, fungicides, rodenticides, algaecides, molluscicides, and nematicides), fertilizers (mainly supplying macronutrients: N, P, and K), soil conditioners (improving the soil’s physical and mechanical qualities), liming (Ca and Mg) and acidifying agents, and plant growth regulators (also known as phytohormones).

With a dramatic increase after the second World War,^1^ the intensive use of agrochemicals has deteriorated the quality of ecosystems (living beings, groundwaters, soils) by impacting human health and, in recent years, leading to the development of pesticide-resistant strains.^2−4^ Over the period of 2011–2018, pesticide sales were around 360,000 tons per year only in the European Union (EU) with the major groups sold being fungicides, herbicides, and bactericides. This is particularly crucial since, according to the Food and Agriculture Organization (FAO) of the United Nations, agriculture occupies about 38% of Earth’s terrestrial surface.^5^ High and repeated doses of hazardous agrochemicals are routinely used to protect crops against pests (insects, fungi, unwanted plants, and others) and boost food productivity (e.g., increasing the number of times per year a crop can be grown on the same territory). With a global population projected to rise above 9.7 billion by 2050, food security is of increasing importance. Herbicides are the most widely used pesticides, comprising >40% of total use, while insecticides and fungicides constitute approximately 30% and 20%, respectively. Pesticide/fertilizer pollution patterns are well-established with a major pollution peak taking place a few days or weeks after agrochemical application.^6^ Ideally, their toxic effect should be limited to both the target area and organisms. However, the lack of specificity of agrochemicals and their widespread use (i.e., in 2018 almost 400,000 tons of pesticides were sold in Europe)^7^ allow them to leach out of the soil and enter surface water and groundwater; therefore, they are even present in drinking water.^8^

On the basis of their application methods, between 10% and 75% of the pesticides do not reach their targets,^9,10^ resulting in frequent contamination of terrestrial and aquatic environments.^9−11^ The EU Drinking Water Directive sets the general drinking water quality standard for added concentrations of pesticides and their metabolites to be less than 0.5 μg·L^–1^. Remarkably, most of the studies in this field report that ∼80% of the studied pesticides are found in concentrations much higher than the EU water quality standard (e.g., 3-fold higher concentrations of tebufenpyrad and pendimethalin in the Louros River in Greece,^12^ 21- and 26-fold higher concentrations of glyphosate and aminomethylphosphonic acid (AMPA) in the area of Zurich, Switzerland,^13^ 40-, 25-, and 20-fold higher concentrations of amitrole, diuron, and terbuthylazine in the Arc River in France, respectively,^14^ and 8-, 12-, 16-, and 25-fold higher concentrations of oxadiazon, pretilachlor, bentazone, and 2-methyl-4-chlorophenoxyacetic acid (MCPA) in the Rhône River in France, respectively,^15^ among others).

Despite the strict EU regulation, pesticides continue enter the food chain through water and food. Regarding other regions, the problem is magnified. For example, it is predicted that in 2050 the major part of global chemical sales will take place in Asia. During the last two decades, South-East Asian countries have shown a strong industrial growth in agriculture.^16^ However, the vast majority of these countries lack the capacity to handle chemical management issues and, furthermore, they still need to develop legislation, institutions, and general awareness. Therefore, this should be considered a global environmental problem. In terms of acute toxicity to humans, many agrochemicals manifest their toxicity through biochemical and functional actions in the central and peripheral nervous system. Also, although not always easy to identify, there is evidence that links long-term exposure to some pesticides with chronic illnesses, including dermal, respiratory, liver, and kidney disorders,^17^ fertility difficulties,^18,19^ postponed neuropathy,^20^ and cancer (e.g., sarcoma, lung, brain, gonads, liver, digestive system, and urinary tract).^20,21^ In this sense, it is likely that the scale and outcome of pesticide-associated chronic effects are underestimated as the symptoms of such poisonings may be incorrectly attributed to other affects. Aside from toxicity to humans, in terms of environmental costs, the unsystematic use of agrochemicals increases pest and disease resistance, diminishes nitrogen fixation and soil biodiversity, and increases the bioaccumulation of pesticides.^22^ Finally, the loss of livestock to resistant bacterial diseases also represents a considerable waste of water and energy investment as well as capital.

Apart from pesticides, fertilizers are among the major contributors to raise crop yield, and therefore, their use has been exponentially enhanced over the past decades (annually >3 million tons have been imported into the EU since 2015).^23^ However, as for pesticides, the use of chemical fertilizers is limited by their poor specificity, increasing both the environmental and production costs (between 50% and 70% of total applied nitrogen is lost by volatilization^24,25^ and 5–10% is lost by leaching).^26^ Further, inefficiencies in the production of food are further intensified by food waste (i.e., ∼33–50% of global manufactured food spoils as consequence of microbial contamination).^27^ The actual scenario of the inefficient use of fertilizers and intensive irrigation, biocides, and processed food is stressing ecosystems and leading to significant environmental collateral injuries (e.g., increasing soil erosion and degradation, loss of biodiversity, rising water withdrawals, reducing water quality, eutrophication, disruption of global nutrient cycles, and increasing the energy consumption and greenhouse gas emissions).^5^ All the above suggests that water, food, nature, and animal and human health are inextricably linked to the agri-food systems.

## 2 Nanotechnology as a Novel Approach in Agrochemical Development

**Figure 1:**
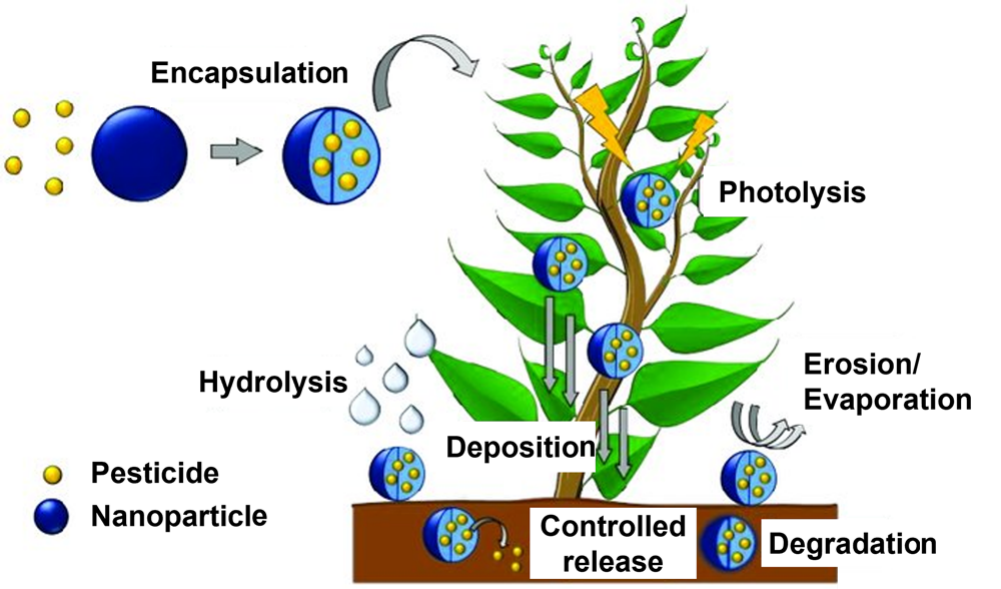
Application of nanotechnology in agriculture. Adapted from ref (39). Copyright 2017 MDPI.

The increase of society’s concern regarding the potential damage of agrochemical application in agricultural production has challenged industry and researchers to search for new efficient and safer methods against insect pests, infections, and unwanted plants or weeds. In this sense, nanotechnology research has recently received an increasing attention in agriculture. With the general aim of developing delivery nanosystems for agrochemicals,^28,29^ nanopesticides and nanofertilizers have been proposed as a novel class of plant protection and growth products that promise a number of benefits to agriculture, the environment and, finally, human health. One of their key drivers is the important reduction in the quantity of agrochemicals necessary to guarantee crop protection and growth, which may be achieved by different ways, such as (i) improved apparent solubility and stability of photo- and thermolabile agrochemicals or active ingredients (AIs), (ii) controlled release and targeted delivery of AIs, and (iii) enhanced bioavailability and adhesion (Figure 1). Nanocarriers of agrochemicals of different natures have been described, including the known “soft” nanoparticles (NPs) (e.g., polymers, lipid, and nanoemulsions) as well as “hard” nanomaterials, such as silica NPs,^30−34^ nanoclays,^35^ TiO_2_,^36^ carbon nanotubes,^37^ or graphene oxides.^38^ Nanocarriers are mainly applied in plant nutrition with the final objective of an increased efficiency of the actually used fertilizers either by enhancing the administration of elements that are poorly bioavailable (P, Zn) or by reducing losses of mobile nutrients to other natural environments (nitrate). However, long-term instability, subsequent burst agrochemical release, and associated toxicity are some of the major drawbacks that need to be addressed.

## 3 Metal–Organic Frameworks as Promising Materials in Agriculture

Among the novel technologies proposed in agriculture, metal–organic frameworks (MOFs) have gained a significant role in the fields of the elimination of agrochemicals (adsorption and/or photodegradation) and sensing. MOFs are considered to be a remarkable class of highly porous coordination polymers, containing inorganic nodes (e.g., atoms, clusters, or chains) and organic linkers (e.g., carboxylates, nitrogenated, or phosphonates), that assemble into multidimensional periodic lattices.^40^ MOFs have been proposed for many societal and industrially relevant applications, such as adsorption,^41^ separation,^42^ magnetism,^43^ luminescence,^44^ conductivity,^45^ sensing,^43^ catalysis,^46^ energy,^47^ drug delivery,^48^ etc. In particular, MOFs are promising materials in agriculture due to their interesting properties: (i) versatile hybrid compositions, which allow a huge variety of combinations, (ii) large specific surface areas and pore volumes, related to exceptional sorption capacities, (iii) simply functionalizable cavities, where specific host–guest interactions may occur, (iv) synthesis at large scale (some of them are already commercialized), and (v) an adequate stability profile, so they are stable enough to accomplish their function and, after being degraded, prevent associated toxicity in animals/plants due to their accumulation.

**Figure 2:**
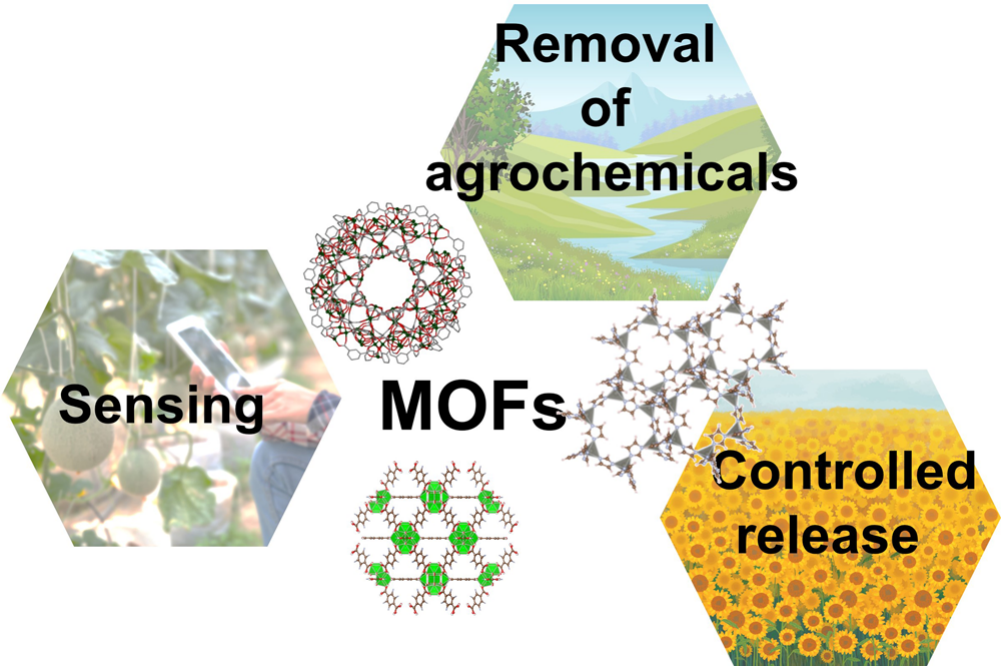
Proposed application related to agrochemicals and MOFs: environmental remediation, controlled release, and detection/quantification of agrochemicals.

Different strategies have been reported in the use of MOF-type materials in agriculture. In particular, related to agrochemicals, MOFs have been proposed (i) in water remediation through the elimination (adsorption/degradation) of agrochemicals or derived products, (ii) as carriers for the controlled release of agrochemicals, and (iii) as sensors for the determination of these molecules in water or food (Figure 2). While not many reviews have detailed the use of MOFs in the elimination of agrochemicals as contaminants in water^49−53^ or their potential in the detection and quantification of these potentially toxic molecules,^51,54,55^ the use of MOFs as agrochemical delivery systems is a very recent research field, initiated in 2015.^56^ Grouped by their function, this Review will discuss the MOFs and MOF-based composites that have been investigated to date in the agricultural domain. In order to give a broad spectrum of benefits and drawbacks of the use of each material, particular features of each structure and its properties are also included. In the text, the most original, interesting, and promising MOFs in agriculture will be highlighted, although all the reports currently found in the literature are summarized in Tables 1 to 3.

**Table 1:** Reported MOFs and MOF Composites Related to the Adsorption and/or Degradation of Agrochemicals^a^

agrochemical	MOF/MOF composite	elimination (% or mg·g^–1^)	conditions	reusability (cycles)	ref, year
acetamiprid	{Sr^II^Cu^II^_6_[(*S*,*S*)-methox]_1.5_[(*S*,*S*)-Mecysmox]_1.50_(OH)_2_(H_2_O)}·36H_2_O	100%	adsorption, 30 s, 100 ppm, aqueous solution	10	(67), 2021
thiacloprid					
alachlor	Cr-MIL-101-C5 (among others)	186.4 mg·g^–1^	adsorption, 24 h, 30 °C, pH = 3–5, 30 ppm, aqueous solutions		(58), 2019
DUR		150.2 mg·g^–1^			
tebuthiuron		95.2 mg·g^–1^			
gramoxone		ca. 60 mg·g^–1^			
ATZ	NU-1000	93%	adsorption, <5 min, 10 ppm, RT, aqueous solutions	3	(61), 2019
ATZ	M.MIL-100(Fe)@ZnO	∼78%	photodegradation, 1 h, 5 ppm, pH = 2, +H_2_O_2_, 500 W Xe, aqueous solutions	5	(68), 2019
ATZ	UiO-67	6.78 mg·g^–1^	adsorption, pH = 6.9, 25 ppm, 2 and 40 min, aqueous solutions		(69), 2018
	ZIF-8	10.96 mg·g^–1^		3	
bentazon	MOF-235	7.15 mg·g^–1^	adsorption in aqueous solutions		(70), 2015
clopyralid		9.76 mg·g^–1^			
IPU		10.00 mg·g^–1^			
chipton	UiO-66-NH_2_@MPCA	227.3 mg·g^–1^	adsorption, 12 h, 10–100 ppm, 30 °C, aqueous solutions	5	(59), 2021
chlorantraniliprole	Al-TCPP	371.91 mg g^–1^	adsorption, 7.5 h, 50 ppm, 25 °C, aqueous solution		(71), 2021
chlorpyrifos	MIL-53(Fe)@AgIO_3_	93–97% (Ad)	adsorption, photodegradation, 1 h, solar light, tap water		(63), 2018
		70% (Photo)			
chlorpyrifos	MIL-53(Fe)@CA	356.34 mg·g^–1^	adsorption, 8 h, 20 ppm, 30 °C, aqueous solution	5	(72), 2021
chlorpyrifos	MIL-53(Fe)@AgIO_3_	78–90%	catalysis, 1 h, solar light, tap water and distilled water		(63), 2018
malathion methyl					
cyhalothrin	ZrO_2_@HKUST-1	99.6%	photodegradation, 6 h, 60 mg·L^–1^, 14 W, 25 °C, aqueous solutions	4	(73), 2019
2,4-D	MIL-53(Cr)	556 mg·g^–1^	adsorption, 1 h, 100 ppm, RT, aqueous solutions	3	(57), 2013
2,4-D	ZIF-8@ionic liquid	448 mg·g^–1^	adsorption, 12 h, 50–200 ppm, pH = 3.5, aqueous solutions		(74), 2017
2,4-DP	[Zn(BDC-NH_2_)(bpd)]	91%	adsorption, 90 min, 60 ppm, water solutions		(75), 2018
2,4-DP	HRP@H-MOF(Zr)	100%	catalysis, 15 min, 6 mM, 25 °C, valley water		(65), 2019
2,4-DP	UiO-66-NMe_3_^+^	279 mg·g^–1^	adsorption, 2 h, 20 ppm, 25 °C, aqueous solutions	7	(76), 2020
2,4-DP	ILCS/U-10	262.45 mg·g^–1^	adsorption, 1 h, pH = 2–4, 25–30 °C, aqueous solutions	4	(77), 2020
diazinon	MIL-101(Cr)	260.4 mg·g^–1^	adsorption, 3 min, 150 ppm, pH = 7, aqueous solution in continuous flow	4	(78), 2018
		92.5%			
diazinon	MIP-202/chitosan–alginate beads	17.77 mg·g^–1^	adsorption, 40 min, 50 ppm, pH = 7, 22 °C, aqueous solution	5	(79), 2021
diazinon	Bp@MIL-125	96%	photocatalysis, 30 min, 20 ppm, pH = 7, UV lamp, aqueous solution		(80), 2021
diazinon	BSA/PCN-222(Fe)	400 mg·g^–1^	adsorption, 3 min, 800 ppm, pH = 7, aqueous solution	12	(81), 2021
parathion methyl		370.4 mg·g^–1^			
dichlorvos	UiO-67	571.43 mg·g^–1^	adsorption, 200 min, 25 °C, 200 ppm, pH = 4, aqueous solutions		(82), 2019
metrifonate		378.78 mg·g^–1^			
		97.8% and 99%			
dimethoate	Cu-BTC@CA	282.3–321.9 mg·g^–1^	adsorption, 6 h, 30 °C, pH = 7, 20 ppm, aqueous solutions	5	(83), 2021
dimethoate	Al-(BDC)_0.5_(BDC-NH_2_)_0.5_	344.7 mg·g^–1^	adsorption, 8 h, 30 °C, 20 ppm, aqueous solutions		(84), 2021
DUR	ZIF-8@ionic liquid	284 mg·g^–1^	adsorption, 12 h, 10–20 ppm, pH = 6.6, aqueous solutions	4	(74), 2017
ethion	CuBTC@Cotton	182 m·g^–1^	adsorption, 2 h, aqueous solutions	5	(85), 2016
		97%			
ethion	ZIF-8	279.3 mg·g^–1^	adsorption, 8 h, 25 °C, 50 ppm, aqueous solutions	4	(86), 2019
	ZIF-67	210.8 mg·g^–1^			
fenamiphos	NU-1000	ca. 6400 mg·g^–1^ (0.89 mol/mol)	adsorption, 2 h, 108.8 ppm, aqueous solution, dynamic conditions	3	(87), 2021
fenitrothion	active-extruded-UiO-66	90.2–95.9%	adsorption, 28 ppm, pH = 7, tap and river water		(88), 2021
fipronil and its metabolites	M-ZIF-8@ZIF-67	95%	adsorption, 1 h, 100 ppm, pH = 6, aqueous solutions and cucumber		(89), 2020
GLU	NU-1000	186 mg·g^–1^	aqueous solutions		(90), 2020
GLY		168 mg·g^–1^			
GLU	UiO-67	360 mg·g^–1^	Adsorption, 300 min, 0.01 mM, 25 °C, pH = 4, aqueous solutions		(91), 2015
GLY	NU-1000	1516.02 mg·g^–1^	adsorption, 20 min, 1.69 ppm, aqueous solutions		(60), 2018
		100%			
GLY	UiO-67	537 mg·g^–1^	adsorption, 300 min, 0.01 mM, 25 °C, pH = 4, aqueous solutions		(91), 2015
GLY	UiO-67@GO	483.0 mg·g^–1^	adsorption, 300 min, pH = 4, 40 ppm, aqueous solutions		(92), 2017
GLY	MIL-101(Cr)-NH_2_	64.25 mg·g^–1^	adsorption, 12 h, 25 °C, pH = 2–4, 100 ppm, aqueous solutions		(93), 2018
GLY	Fe_3_O_4_@SiO_2_@UiO-67	256.54 mg·g^–1^	adsorption, 2 h, RT, 20–70 ppm	4	(94), 2018
imidacloprid	Bi_2_WO_6_/MIL-88B(Fe)-NH_2_	84%	photocatalysis, 3 h, 10 ppm, pH = 9, Xe lamp	5	(95), 2021
IPU	CPO@H-MOF(Zr)	100%	catalysis, 15 min, 20 μM, 25 °C, valley water		(65), 2019
mecoprop	UiO-66	51 mg·g^–1^	adsorption, 6 h, 20–170 ppm, 25 °C, pH = 2–5, aqueous solutions	3	(96), 2015
mecoprop	Basolite Z1200		adsorption, aqueous solutions		(97), 2013
NIT	PCN-224	95%	photodegradation, 20 min, aqueous solution		(62), 2020
paraquat	MIL-101(Cr)@α-Fe_2_O_3_@TiO_2_	87.5%	catalysis, 45 min, 20 ppm, pH = 7, 25 °C, aqueous solutions		(64), 2018
paraoxon	UiO-66	100%	catalysis, 30 min, RT, 1 mM, pH = 7.8, aqueous solutions		(98), 2018
parathion methyl	CuBTC@PAN	90%	adsorption, 2 h, aqueous solutions		(99), 2014
propiconazole	MIL-101(Cr)	89.3%	adsorption, 100 min, pH = 3, aqueous solutions	5	(100), 2021
prothiofos	ZIF-8	366.7 mg·g^–1^	adsorption, 8 h, 25 °C, 50 ppm, aqueous solutions	4	(86), 2019
	ZIF-67	261.1 mg·g^–1^			
QPE	QpeH@ZIF-10	88%	enzymatic degradation, 14 days, pH = 6.7, watermelon field	10	(66), 2021
thiamethoxam	MIL-100(Fe)@Fe-SPC	95.4%	catalysis, 180 min, 60 ppm, pH = 7.5, 25 °C, +H_2_O_2_, us	5	(101), 2018
NND	M-MOF	1.8–3.0 mg·g^–1^	adsorption, 1 h, 100 ppm, aqueous mixture of contaminants		(102), 2017
OP	ZIF-8@M-M	96%	adsorption, 15 min, 0.2–8 ppm, pH = 2–10, aqueous mixture of contaminants	5	(103), 2018

a The table is sorted according to the studied agrochemical, followed by the MOF-based material name (or chemical formula), elimination capacity (% or mg·g^–1^), optimal conditions for the elimination (mechanism, time to reach the equilibrium, concentration of the agrochemical, temperature, pH, type of light, and other species involved during the catalytic process), and cycles of reuse. Bp: black phosphorus; bpd: 1,4-bis(4-pyridyl)-2,3-diaza-1,3-butadiene; BSA: bovine serum albumin; CA: cellulose acetate; Fe-SPC: Fe-doped nanospongy porous biocarbon; GO: graphene oxide; H_2_BDC-NH_2_: 2-aminoterephthalic acid; HRP: horseradish peroxidase; H_3_BTC: 1,3,5-benzenetricarboxylic acid; ILCS: ionic liquid modified chitosan; M-M: magnetic multiwalled carbon nanotubes; MPCAs: carbon nanotube aerogels; PAN: polyacrylonitrine; QpeH: quizolafop-P-ethyl hydrolase esterase; RT: room temperature; us: ultrasound.

## 4 MOFs in Environmental Remediation: Agrochemicals Elimination

**Figure 3:**
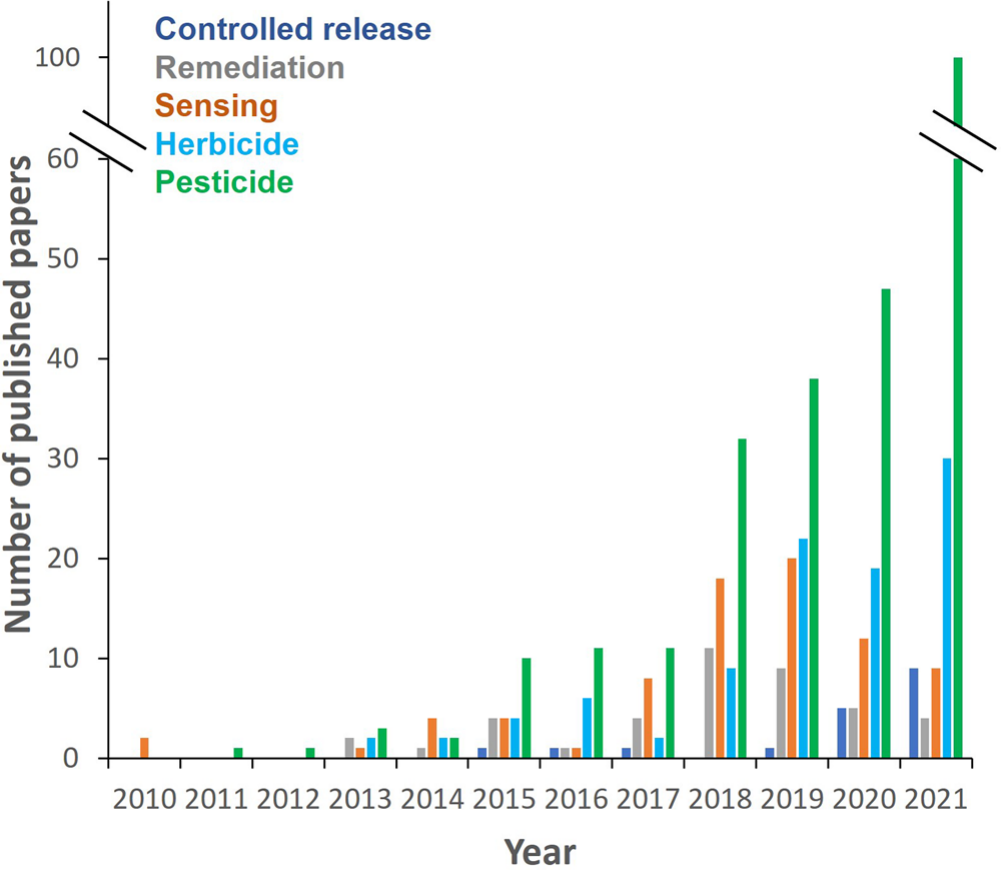
Number of published papers having keywords MOF and agriculture in their titles and abstracts, separated by areas (i.e., controlled release, remediation, and sensing) and important related words (herbicide and pesticide). Retrieved from the Web of Science on March 10, 2022.

In recent years, MOFs have exponentially been investigated for the removal (mainly adsorption or degradation processes) of different contaminants from water. Originally, this field was focused on the removal of organic dyes, although recently, agrochemicals have also been included as target contaminants due to their increasing presence in natural waters and their severe toxicity to living beings. Thus, an increase in the number of reports dealing with the elimination of different agrochemicals using MOFs has been reported (Figure 3), including (i) **herbicides**: alachlor, atrazine (ATZ), bentazon, chipton, clopyralid, 2,4-dichlorophenoxyacetic acid (2,4-D), diuron (DUR), glufosinate (GLU), glyphosate (GLY), gramoxone, isoproturon (IPU), mecoprop, paraquat, quizalofop-P-ethyl (QPE), and tebuthiuron; (ii) **fungicides**: propiconazole and thifluzamide (THI); (iii) **insecticides**: chlorantraniliprole, chlorpyrifos, cyhalothrin, diazinon, dichlorvos, dimethoate, ethion, fenamiphos, fenitrothion, malathion methyl, metrifonate, nitenpyram (NIT), paraoxon, parathion methyl, prothiofos, thiamethoxam, several neonicotinoids (NND, acetamiprid, clothianidin, dinotefuran, imidacloprid, NIT, thiacloprid, and thiamethoxam), and organophosphorus pesticides (OP, diazinon, ethoprop, isazofos, methidathion, phosalone, profenofos, sulfotep, and triazophos). Table 1 summarizes the reported studies regarding agrochemical removal using MOFs and MOF-based composites with a summary of the conditions and results of each study (on the basis of the reported data presented by the authors).

### 4.1 Adsorption Processes

**Figure 4:**
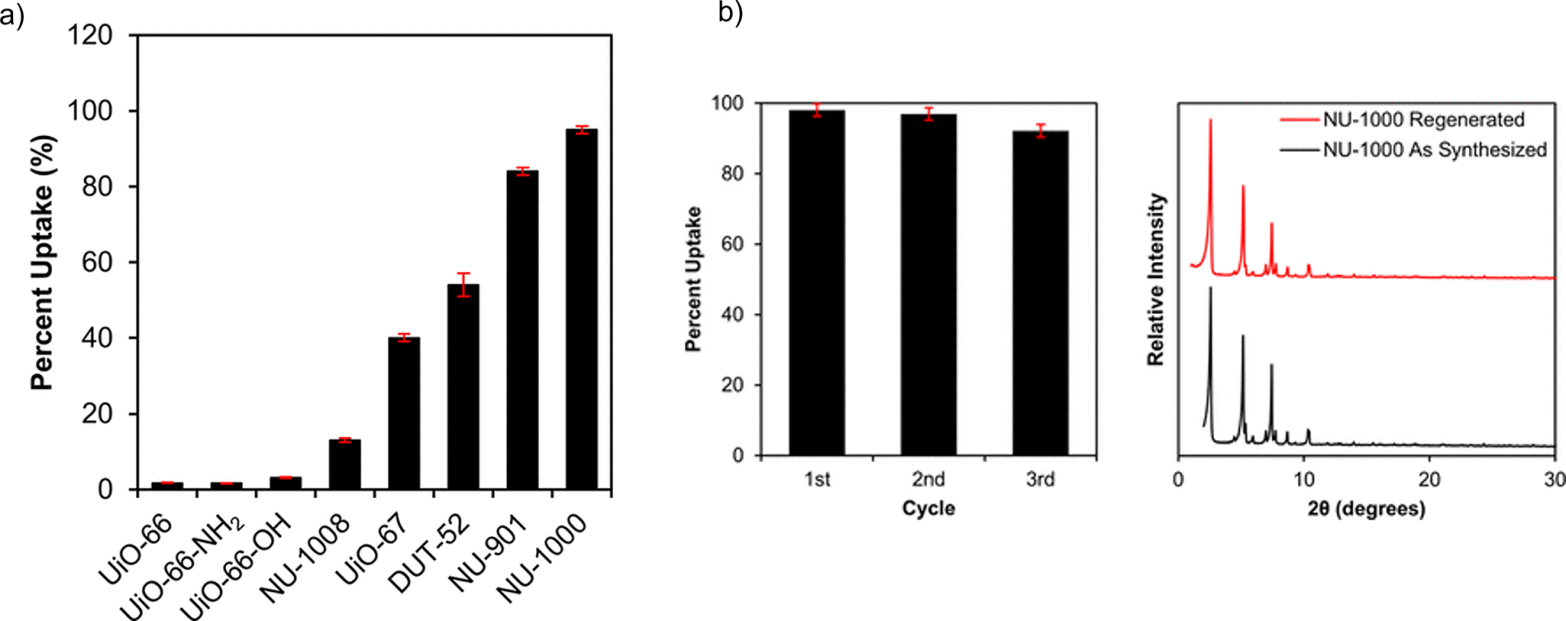
(a) ATZ uptake as a percentage of the total amount of ATZ exposed to Zr-MOFs for 24 h and (b) through 3 cycles of ATZ adsorption and regeneration with acetone using NU-1000 while maintaining its crystallinity. Reprinted from ref (61). Copyright 2019 American Chemical Society.

Regarding the adsorption of herbicides, the first study was published by Jung et al. in 2013 for the removal of 2,4-D using the MIL-53(Cr) material ([Cr(OH)(BDC)]; H_2_BDC: benzene-1,4-dicarboxylic acid, pore size of ∼8 Å).^57^ MIL-53(Cr) exhibited an efficient and fast adsorption (556 mg·g^–1^ in 1 h) with an adsorption capacity much higher than that of activated carbon (286 mg·g^–1^) or zeolite (256 mg·g^–1^). Importantly, the adsorption of 2,4-D at a very low concentration is 5-fold greater that of activated carbon at a plateau concentration, demonstrating the utility of MIL-53(Cr) in commercial uses for consumed water with low 2,4-D levels. Finally, the recyclability of MIL-53(Cr), after washing the MOF with a mixture of water/ethanol, was also noticeable after 3 cycles, suggesting the potential application of this MOF on the herbicide’s removal. In a more recent work, a series of furan-thiophene derived from Cr-MOF MIL-101(Cr) ([Cr_3_(O)X-(BDC)_3_(H_2_O)_2_], X = OH or F; Brunauer–Emmett–Teller surface area (*S*_BET_) ∼ 4100 m^2^ g^–1^; pore volume (*V*_p_) = 2.02 cm^3^ g^–1^; pore size of 11.7 and 16 Å) was achieved via visible-light-mediated C–C bond-forming catalysis within photosensitizing porous materials.^58^ The process of trapping the guest molecule was accomplished under metal-free and very mild conditions, leading to novel functionalized MOFs with more π–π stacking, H bonding properties, and outstanding adsorption capacity to eliminate herbicides from the aqueous solutions. The synthesized MOFs removed up to 96.9% of the tested herbicides from the aqueous solutions even at initially very low herbicide concentrations (30 ppm). Particularly, the Br derivative MIL-101(Cr)-C5 inhibited the maximum adsorbed capacities for DUR, alachlor, and tebuthiuron with adsorption capacities of 186.4, 150.2, and 95.2 mg·g^–1^, respectively. Very recently, a composite based on Zr-MOF UiO-66-NH_2_ ([Zr_6_O_4_(OH)_4_(BDC-NH_2_)_6_]·*n*H_2_O; *S*_BET_ ∼ 950 m^2^ g^–1^; pore size of ∼11 and 8 Å; H_2_BDC-NH_2_: 2-aminoterephthalic acid) was described for the removal of herbicides in water.^59^ UiO-66-NH_2_ was loaded on the carbon nanotube aerogels (MPCAs) by the *in situ* nucleation and growth of the UiO-66-NH_2_ NPs onto the carbon nanotubes (UiO-66-NH_2_@MPCA). The study on the adsorption of chipton and alachlor demonstrated that the adsorption capacity of UiO-66-NH_2_@MPCA was improved with respect the single MOF NPs, which is indicative of a synergistic effect between the MOF and MPCA (i.e., the chipton adsorption capacity is improved from 98.4 to 227.3 mg·g^–1^ for UiO-66-NH_2_ and UiO-66-NH_2_@MPCA, respectively). Further, rice was used to assess the biosecurity of the composite. Remarkably, UiO-66-NH_2_@MPCA could reduce the accumulation of Zr^4+^ in the roots and leaves of rice in comparison with the UiO-66-NH_2_ NPs, demonstrating that MPCA can diminish the potential environmental risk of the MOF materials. Lastly, the authors demonstrated the reusability of the composite up to 5 times without decreasing its adsorption capacity. Finally, we want to highlight two reports utilizing the water stable Zr-based MOF NU-1000 ([Zr_6_(μ_3_-O)_4_(μ_3_–OH)_4_(−OH)_4_(−OH_2_)_4_(PyTBA)_2_]; PyTBA: 4,4′,4″,4‴-(pyrene-1,3,6,8-tetrayl)tetrabenzoate; *S*_BET_ ∼ 2100 m^2^·g^–1^; pore size of ∼12 and 30 Å). In the first study, reported by Pankajakshan et al.,^60^ NU-1000 was described for the efficient elimination of GLY from aqueous media. NU-1000 comprises [Zr_6_(μ_3_-O)_4_(μ_3_-OH)_4_(H_2_O)_4_(OH)_4_] as secondary building units, acting as Lewis acid nodes that can react with the Lewis base phosphate group of GLY. Theoretical calculations demonstrated that the interaction energy of GLY with the NU-1000 nodes was −37.63 KJ·mol^–1^. NU-1000 was synthesized in different particle size scales (100–2000 nm), reducing the equilibrium times with smaller MOF sizes and achieving a total GLY loading of 1516.02 mg·g^–1^ (or 8.97 mg·g^–1^) in only 20 min. In the second work, the same authors thoroughly investigated the mechanism governing ATZ adsorption on Zr_6_-based MOFs (UiO-66-X, where X = H, OH, NH_2_; DUT-52; UiO-67; NU-901; NU-1000; and NU-1008) by investigating the impact of MOF used linkers and topology on ATZ uptake capacity and kinetics.^61^ Among all the tested Zr-MOFs, it was found that the mesopores of NU-1000 facilitate the rapid ATZ uptake, saturating in less than 5 min. Excluding the pyrene-based linker, NU-1008 ([Zr_6_(μ-O)_4_(μ-OH)_4_(HCOO)(H_2_O)_3_(OH)_3_(TCPB)_2_]; TCPB: 1,2,4,5-tetrakis(4-carboxyphenyl)benzene; *S*_BET_ ∼ 1400 m^2^·g^–1^; pore size of ∼14 and 30 Å) removed <20% of the exposed ATZ. The pyrene-based linker seems to offer enough sites for π–π interactions with ATZ as revealed by the near 100% uptake (Figure 4). These results indicate that the ATZ uptake in NU-1000 stems from the existence of a pyrene core in the linker of the MOF, which confirms that the π–π stacking is the main force of the ATZ adsorption. Finally, the cyclability of the MOF was demonstrated through 3 adsorption–desorption cycles.

### 4.2 Catalytic Processes

The number of studies related to the catalytic degradation of agrochemicals using MOFs or MOF composites are very limited. The literature mainly focuses on the use of MOF composites with only one work based on a simple MOF. This study describes the bifunctional nanoscale porphyrinic MOF PCN-224 (or [Zr_6_(TCPP)_1.5_]; H_2_TCPP: tetrakis(4-carboxyphenyl)porphyric; *S*_BET_ = 2600 m^2^·g^–1^; *V*_p_ = 0.95 cm^3^·g^–1^; pore size = 1.9 nm) as both the sensor for the recognition of trace NIT and the photocatalyst to enable the pesticide degradation.^62^ The intense fluorescence of the probe was quenched by NIT, leading to a sensing range from 0.05 to 10.0 μg·mL^–1^. The potentiality of PCN-224 in the degradation of NIT was further identified. The photodegradative effectiveness was up to 95% after only 20 min of laser irradiation, whereas no significant NIT degradation was detected under darkness, regardless of the PCN-224 presence. Therefore, this material could be established as an all-in-one nanoplatform for pesticide sensing, detection, and posterior photodegradation in agricultural farmland and other environments. Among other MOF-based composites, metallic NP composites are the most employed. The MIL-53(Fe)@AgIO_3_ composite was successfully applied in the decomposition of two organophosphate pesticides (OPs; malathion methyl and chlorpyrifos) under sunlight irradiation.^63^ After 1 h of solar light irradiation, 78–90% of both pesticides was individually degraded in tap and distilled water. In binary mixtures (both composites), 70% of mineralization is achieved after 3 h. Another example of metallic NPs@MOF composites is magnetic α-Fe_2_O_3_@MIL-101(Cr)@TiO_2_ for the degradation of paraquat herbicide from aqueous solution.^64^ A maximum photocatalytic degradation was achieved at optimal conditions (see Table 1; 87.5% after 45 min), demonstrating the utility of these systems in the photocatalytic degradation of agrochemicals in water.

MOFs have also been used as support for the stabilization of enzymes able to degrade agrochemicals in wastewater and soil. Gao et al. described the preparation of a hierarchically porous MOF (H-MOF(Zr)) as support of the chloroperoxidase (CPO) and horseradish peroxidase (HRP) enzymes, leading to the CPO/HRP@H-MOF(Zr) composite.^63^ CPO@H-MOF(Zr) and HRP@H-MOF(Zr) composites were applied in the treatment of wastewater containing IPU and 2,4-D, achieving a complete and very fast (15 min) degradation. Finally, we want to highlight the fabrication of purified esterase embedded in zeolitic imidazolate frameworks (ZIFs) for the degradation of pesticides.^66^ Particularly, in this work, aryloxyphenoxypropionate herbicide-hydrolyzing enzyme, QpeH, was embedded into ZIF-10 ([Zn(Im)_2_]; Im: imidazonate) and ZIF-8 ([Zn(Hmim)_2_]; Hmim: 2-methylimidazole; *S*_BET_ ∼ 1260 m^2^ g^–1^, *V*_p_ ∼ 0.6 cm^3^ g^–1^, pore size of ∼3.4 and 11.4 Å) and tested in the degradation of quizalofop-P-ethyl in a watermelon field. Remarkably, the QpeH@ZIF-10 composite showed a slightly improved degradation efficiency compared to QpeH@ZIF-8 (88% vs 84%). Unfortunately, no ZIF degradation studies were performed in this research in an attempt to rationalize the different behaviors of both ZIF composites and their potential application in real water treatments. It should be noted that the QpeH@ZIF composites were demonstrated to affect the recovery of the bacterial community in soil.

## 5 MOFs as Agrochemical Delivery Agents

The recent enthusiasm around the use of MOFs as agrochemical delivery agents is highlighted in Figure 3 with a significant increase in the number of papers published on the topic in the last two years. All the studies reported so far on the controlled release of agrochemicals from MOFs are summarized in Table 2, again sorted by the different types of released agrochemical: (i) **herbicides**, *cis*-1,3-dichloropropene (1,3-DCPP) and ortho-disulfides; (ii) **fungicides**, diniconazole, prochloraz, tebuconazole, and zoxystrobin; (iii) **insecticides**, chlorantraniliprole, λ-cyhalothrin, dinotefuran, imidacloprid, and thiamethoxam; (iv) **fertilizers**: urea; (v) **plant grow regulators**: gibberellin. The first MOF described as a delivery agent of agrochemicals was OPA-MOF (OPA: oxalate–phosphate–amine). In 2015, Anstoetz et al. described the use of an OPA-MOF as a microbially induced slow-release N and P fertilizer.^56^ In this research, the capacity of the urea-templated OPA-MOF as a new fertilizer with a slow release was investigated and compared with a standard P (triple superphosphate) and N (urea) fertilizer (ferralsol). The authors hypothesize that the OPA-MOF is a gradual-release fertilizer for crops grown on acidic soils, where microbial consumption of the oxalate linker gives rise to the degradation of the framework structure, thereby releasing Fe phosphate. While in the OPA-MOF treatment the hydrolysis of urea was fast, the conversion of the ammonium to nitrate was significantly diminished in comparison with the urea treatment (ferralsol). However, P uptake and yield in OPA-MOF was considerably lower than in conventionally fertilized plants. OPA-MOF was proven to have potential as an enhanced efficiency N fertilizer but not in P bioavailability. A year later, two novel OPA-MOFs were hydrothermally synthesized and fully characterized to be used again as slow-release fertilizers.^104^ The framework backbone is robust and based on FeO_6_ units with bidentate oxalate bridges joining adjacent Fe centers. PO_4_ units have corner-sharing for all of their oxygens with the FeO_6_ units. The authors studied the release of oxalate, setting it high enough to permit oxalate concentrations in the soil solution to achieve 1 mg·L^–1^ but also low enough to avoid fast and purely chemically driven compound degradation. The results show that, from the two synthesized materials, OPA-MOF-I has a slow solubility with an oxalate concentration of ca. 5 mg·L^–1^ at high loading and seems to be compatible with trials as a fertilizer in future works.

**Table 2:** Previously Reported MOF-Based Materials Associated with the Controlled Release of Agrochemicals^a^

agrochemical	MOF/MOF composite	loading (wt % or mmol·g^–1^)	release conditions	activity studies	ref, year
azoxystrobin	MIL-100(Fe)	16.2 wt %	80% (pH = 5.0), 85% (pH = 7.2), 86% (pH = 8.5) with PBS, ethanol, and Tween-80 emulsifier	fungicidal activity against (*Fusarium graminearum* and *Phytophthora infestans*) 5 and 15 ppm, 56% and 62% of inhibition in 7 days, nutritional function of Fe	(109), 2020
azoxystrobin; diniconazole	MIL-101(Al)-NH_2_	6.71%; 29.72%	90% in 46 and 136 h	germicidal efficacy against rice sheath blight (*Rhizoctonia solani*), EC_50_ = 0.065 mg·mL^–1^	(110), 2021
λ-cyhalothrin	UiO-66	87.71 wt %	70% in 12 h in DMF or 60% DMF aqueous solution	insecticide activity assay (*Musca domestica*) KT_50_ = 3.64, 5.12, and 6.91 min after being treated 1, 15, and 30 days; bioactivity (*Aphis craccivora*) LC_50_ = 3.20, 0.70, and 0.36 ppm at 24, 48, and 72 h	(111), 2020
1,3-DCPP	MOF-1201; MOF-1203	1.4 mmol·g^–1^; 13 wt %	80% in 100 000 min·g^–1^ under air flow 1.0 cm^3^·min^–1^		(105), 2017
chlorantraniliprole	MIL-101(Fe)@silica	23%	dialysis method, water, sink conditions	photostability improvement (16.5 times more stable), insecticidal activity against *Plutella xylostella* (LC_50_ = 0.389 mg·L^–1^)	(112), 2021
diniconazole	PDA@NH_2_-Fe-MIL-101	28.1 wt %	PBS/ethanol/Tween-80 emulsifier	fungicidal activity against *Fusarium graminearum* 1 and 5 ppm for 4 days of inhibition of 44% and 86%	(113), 2020
dinotefuran	MIL-101(Fe)@CMCS	24.5%	83.1% aqueous solution stimulated by citric acid in ca. 18 h	photostable (70%) after 48 h of irradiation, insecticidal activity in soil	(114), 2020
dinotefuran, Zn^2+^	PFAC	13.60%	photothermal triggered release (49% at 40 °C), pH response release (pH = 4.0 and 7.0 is 52.63% and 31.87%)	stem length (39.2 vs 33.9 cm) and root length (19.7 vs 13.5 cm) of the corn were clearly improved after 25 days of cultivation	(115), 2021
gibberellin	CLT6@PCN-Q	0.78 mmol g^–1^	release under stimuli (pH, temperature, and competitive agent)	germination of Chinese cabbages and monocotyledonous wheat	(116), 2021
imidacloprid	Fe_3_O_4_@PDA@UiO-66	15.87%	dialysis method in water (50% in 48 h)	insecticidal activity against *Aphis craccivora* Koch (LC_50_ = 2.15 mg·L^–1^, comparable to the commercialized formulation)	(117), 2021
NH_4_^+^	MOF(Fe)@NaAlg(2:10)	1.63 mmol·g^–1^	release in water (80%) and soil (69%) in 28 days	water retention of soil	(118), 2020
ortho-disulfides (DiS-NH_2_ and DiS-*O*-acetyl)	ZIF-8	42.8 and 16.71 wt %	ca. 85% in 2 h of PBS (pH = 5.5)	IC_50_ = 5.413 and 3.892 μM, phytotoxicity bioassay against *Echinochloa crusgalli*, *Amaranthus viridis*, and *Lollium rigidum*	(119), 2021
oxalate; urea	OPA-MOF	3.1% of N; 12.5% of P, 14.5% of oxalate		soil incubation and crop growth (wheat)	(56), 2015
oxalate; urea	OPA-MOF I and II	3.2% and 5.8% of N; 11.3% and 15.6% of P			(104), 2016
prochloraz	PD@ZIF-8		pH and light response, release in dark (13.7%) vs light (63.4%)	cytotoxicity under light EC_50_ = 0.122 μg·mL^–1^, fungal activity (*Sclerotinia sclerotiorum*), updated in plants (oilseed rape)	(120), 2021
tebuconazole	MIL-101(Fe)-TA	24.1 wt %	stimuli response (pH, sunlight, H_2_O_2_, GSH, PO_4_^3–^, and EDTA)	cytotoxicity (HLF-1), safety (wheat seedlings), and fungicidal activity (*Rhizoctonia solani* and *Fusarium graminearum*)	(107), 2021
tebuconazole	PCN-224@P@C	30 wt %	174 h in PBS solution (pH = 5) 17.2%, stimuli response to pectinase in PBS (pH = 5) 86.9% in 174 h	fungicidal activity (*Xanthomonas campestris* pv *campestris*, *Pseudomonas syringae* pv *tomato*, and *Alternaria alternate*) and safety (Chinese cabbage)	(106), 2019
thiamethoxam	UiO-66-NH_2_/SL	33.56 wt %	PBS solution at 37 °C (ca. 80% in 60 h), soil column (76.8% in 48 days)	biosafety (100% rice seed germination)	(121), 2021
TMPyP	HKUST-1		light irradiation (day/night temperature was 25/18 °C, photoperiod was 15/9 h, and the humidity was at 60–80% (irradiance of 9 mW cm^–2^ and energy of 3.18 kJ cm^–2^)	photodynamic fungicidal activity (*P. syringae pv lachrymans* and *C. michiganense* subsp. *Michiganense*), efficacy (*Sclerotinia sclerotiorum*), and safety to plants (cucumber and Chinese cabbage)	(108), 2021

a The table is organized according to the agrochemical, followed by the MOF-based material name (or chemical formula), loading capacity (wt % or mmol·g^–1^), release conditions, and activity tests. C: chitosan; CMCS: carboxymethyl chitosan; DMF: *N*,*N′*-dimethylformamide; EDTA: ethylenediaminetetraacetate; GSH: glutathione; HLF-1: human lung fibroblast; IC_50_: half maximal inhibitory concentration; KT_50_: 50% knockdown time; LC_50_: median lethal concentration; OPA: oxalate-phosphate-amine; P: pectin; PBS: phosphate buffer saline; PD: prochloraz (P) and 2,4-dinitrobenzaldehyde (D); PDA: polydopamine; SL: sodium lignosulfonate; TA: tannic acid; TMPyP: 5,10,15,20-tetrakis(1-methyl-4-pyridinio)porphyrin tetra(*p*-toluenesulfonate).

**Figure 5:**
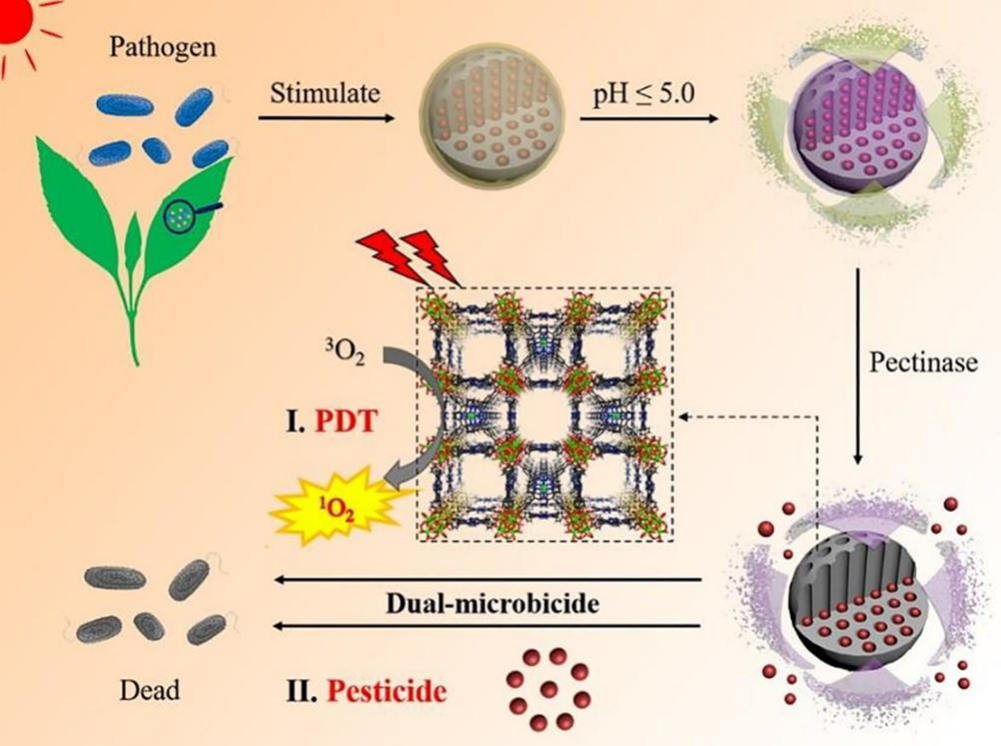
Mechanism of triggered tebuconazole release and the illustration of the dual-microbicidal effect of the Tebuc@PCN@P@C microcapsules. Reprinted from ref (106). Copyright 2021 American Chemical Society.

In 2017, Yaghi and co-workers described a naturally degradable MOF as a carrier of the important fumigant *cis*-1,3-dichlorophropene (1,3-DCPP).^105^ The MOF [Ca_14_(l-lactate)_20_(acetate)_8_X] (X: C_2_H_5_OH, H_2_O, or MOF-1201), constructed from Ca^2+^ ions and l-lactate, presents apertures and an internal diameter of 7.8 and 9.6 Å, respectively, and a permanent porosity of 430 m^2^·g^–1^. MOF-1201 can efficiently encapsulate the 1,3-DCPP agrochemical with a total pesticide loading of 1.4 mmol·g^–1^ (13 wt %). Originally, the fumigant release study was performed using an air flow, demonstrating a slow release when purging samples of the 1,3-DCPP loaded MOF (1,3-DCPP@MOF-1201) in an air flow of 1.0 cm^3^·min^–1^. The loaded 1,3-DCPP@MOF-1201 showed a 100 times slower release compared to that of the liquid 1,3-DCPP, achieving 80% of the total release in 100 000 min·g^–1^. Porphyrinic MOFs have also been described as promising carriers of fungicides.^106^ Particularly, PCN-224 was loaded (30 wt %) with tebuconazole and constructed layer by layer with chitosan and pectin to get tebuconazole microcapsules. The synthesized microcapsules (Tebuc@PCN@P@C) had a dual-microbial effect on plant bacterial and fungal diseases (Figure 5). First, the tebuconazole previously loaded in the microcapsules was gradually released (87% in 7.25 days) after the pectin layer was decomposed by the pectinase released by the invading pathogen. Second, the singlet oxygen (^1^O_2_) was released from the organic linker porphyrin when the MOF NPs were exposed to light after the formation of pectin to inhibit the pathogens. The synthesized compound displayed excellent double activities of having photodynamic therapy and being microbicidal against the bacteria *X. campestris* pv *campestris* (82.4% and 18.4% under light and dark, respectively) and *P. syringae* pv *tomato* (56.3% and 9.5% under light and dark, respectively) and the fungi *A. alternate* (68.0%). Finally, the authors studied the safety of this compound against Chinese cabbage (*Brassica rapa pekinensis*) in a greenhouse environment. The results demonstrated that the synthesized microcapsules do not have a major effect on both the fresh weight and the soil plant analysis development (SPAD) value of the tested plant leaf, so the Tebuc@PCN@P@C microcapsules can be considered safe.

**Figure 6:**
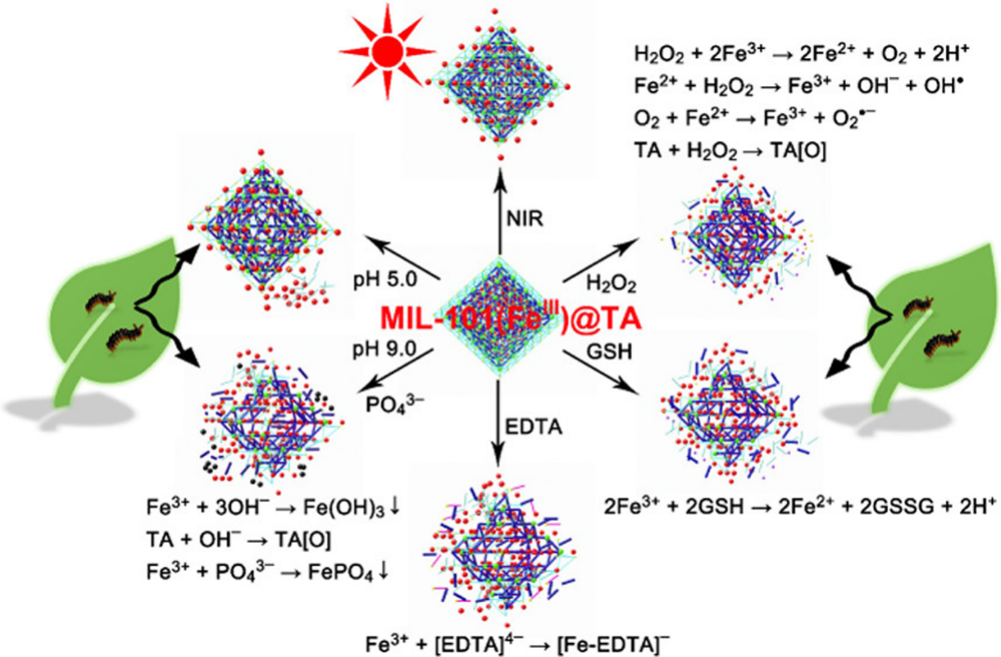
Stimuli-responsive controlled release in MIL-101(Fe^III^) nanopesticides gated with Fe^III^-TA networks related to the biological and natural environments of crops and stimuli-responsive mechanisms. TA[O] represents the oxidation product of TA. Reprinted with permission from ref (107). Copyright 2020 Elsevier Inc.

A very recent and complete study described a further example of a tebuconazole loaded MOF, the MIL-101(Fe) gated with Fe^III^-tannic acid (TA) networks.^107^ The Fe^III^-TA complexes are able to absorb UV–vis near-infrared (NIR) lights. The design of MIL-101(Fe)-TA NPs enables the release of the tebuconazole cargo (24.1 wt %) in response to 7 stimuli (i.e., acidic pH, alkaline pH, H_2_O_2_, glutathione (GSH), phosphate, ethylenediaminetetraacetate (EDTA), and sunlight) to meet the diverse controlled release of the encapsulated cargo (Figure 6). Tebuconazole is gradually released from the gated MIL-101(Fe) when the pH decreases to 5.0 as a result of the partial disassembly of Fe^III^-TA networks, and a significant delivery of the pesticide occurred when the pH increases to 9.0 owing to both the disassembly of the Fe^III^-TA networks and the degradation of the MIL-101(Fe). This is important since, in various parts of the plants themselves or caused by pest and pathogens, there are different pH values. Further, when crop plants suffer from biotic or abiotic stress, H_2_O_2_ is rapidly produced in cells. On the basis of the Fenton reaction between H_2_O_2_ and Fe^II^/Fe^III^, the release of the cargo will be induced by the degradation of MIL-101(Fe). GSH, normally found in plants and animals, is able to reduce Fe^III^ to Fe^II^, causing the degradation of MIL-101(Fe) and, then, promoting the release of the encapsulated pesticide. Additionally, phosphates can induce MIL-101(Fe) degradation by competitive coordination with Fe^III^, and finally, the Fe^III^-TA networks on MIL-101(Fe) will stimulate the controlled release of the pesticide via the photothermal effect of the NIR light of sunlight. Lastly, this system demonstrated high fungicidal activities against *R. solani* (rice sheath blight; concentration for 50% of the maximal effect, ED_50_: 0.4960 mg·L^–1^ after 48 h) and *F. gaminearum* (wheat head blight; ED_50_: 0.5658 mg·L^–1^ after 48 h); good safety in seed germination, seedling emergence, and plant height of wheat by seed dressing; satisfactory control efficacies on wheat powdery mildew caused by *B. graminis*.

Finally, we want to mention a report on the construction of a porphyrin MOF nanocomposite constructed by incorporating 5,10,15,20-tetrakis(1-methyl-4-pyridinio)porphyrin tetra(*p*-toluenesulfonate) (TMPyP) as a photosensitizer (PS) in the cage of HKUST-1 (or CuBTC, [Cu_3_(btc)_2_(H_2_O)_3_]) (HKUST: Hong Kong University of Science and Technology, *S*_BET_ ∼ 1300–1600, *V*_p_ ∼ 0.71 cm^3^·g^–1^) to efficiently produce singlet oxygen to inactivate plant pathogens under light irradiation.^108^ The prepared PS@HKUST-1 loaded about 12 wt % of PS, exhibiting an excellent and broad-spectrum photodynamic antimicrobial activity *in vitro* against three plant pathogenic fungi (*S. sclerotiorum*, *P. aphanidermatum*, and *B. cinerea* with >80%, 60%, and 80% efficiency at concentrations of 200, 50, and 200 mg·L^–1^, respectively) and two pathogenic bacteria (*P. syringae* pv *lachrymans* and *C. michiganense* subsp. *Michiganense*). Besides, *Allium cepa* chromosome aberration assays confirmed that PS@HKUST-1 showed no genotoxicity and safety to the growth of cucumber and Chinese cabbage.

Thus, considering all the mentioned examples, the controlled release of agrochemicals from MOFs and MOF composites is an emerging research field that has demonstrated a great potential as an alternative and efficient new strategy to release plant nutrients but also control pests in agricultural applications.

## 6 MOFs as Sensors of Agrochemicals

Considering the important detrimental effect of pesticides on human health, researchers have extensively studied and discussed the pretreatment, extraction, detection, and determination of agrochemical residues in water, fruits, and vegetables. These data have been valuable to food analysts and regulatory authorities for monitoring the quality and safety of fresh food products, among others. As would be expected, MOFs and MOF composites have been extensively proposed for the extraction and determination of these dangerous substances in food and water. Many different agrochemicals have been analyzed using MOFs: (i) **herbicides**, ametryn, amidosulfuron, ATZ, atraton, bensulfuron methyl, bis(*p*-nitrophenyl) phosphate, butachlor, carbaryl, chlorimuron ethyl, 2,4-dichlorophenol, 4-chlorophenoxyacetic acid (4-CPA), chlortoluron, 2,4-D, dicamba, desmetryn,2-(2,4-dichlorophenoxy)propionic acid (2-DPP), chlorotoluron, dipropetryn, epoxide, fenuron, fluometuron, glufosinate, GLY, heptachlor prometryn, MCPA, (2-methyl-4-chlorophenoxy) butyric acid (MCPB), (2-methyl-4-chlorophenoxy) propionic acid (MCPP), metsulfuron methyl, monuron, nicosulfuron, metazachlor, molinate, nitrofen, paraquat, pretilachlor, prometon, propanil, pyrazosulfuron ethyl, secbumeton, simazine, sulfometuron methyl, sulfosulfuron, terbumeton, terbuthylazine, thifensulfuron methyl, trietazine, and trifluralin; (ii) **fungicides**: bromuconazole, carbendazim, chlorothalonil, 2,6-dichloro-4-nitroaniline (2,6-DN), difenoconazole, diniconazole, epoxiconazole, fenbuconazole, flusilazole, flutriafol, hexachlorobenzene, hexaconazole, iprodione, myclobutanil, penconazole, prochloraz, propiconazole, pyraclostrobin, pyrimethanil, tebuconazole, thiabendazole, thiophanate methyl, and triadimefon; (iii) **insecticides**: aldrin, avermectin, azinphos methyl, bifenthrin, bromopropylate, carbofuran, chlordane, chlorfluazuron, chlorpyrifos, clethodim, clofentezine, coumaphos, β-cyfluthrin, cyhalothrin, cyphenothrin, deltamethrin, diazinon, *o*,*p*′- and *p*,*p*′-1,1-dichloro-2,2-bis(*p*-chlorophenyl)ethylene (DDE), *p*,*p*′-1,1-dichloro-2,2-bis(*p*-chlorophenyl)ethylene (*p*,*p*′-DDD), dichlorvos, dieldrin, difenoxuron, diflubenzuron, dimethoate, diniconazole, dinotefuran, α- and β-endosulfan, fenitrothion, fenpropathrin, fenthion, fenvalerate, flufenoxuron flumetralin, α-, β-, γ-, and δ-hexachlorocyclohexane (HCH), hexaflumuron, imidacloprid, isocarbophos, lufenuron, malathion (also named carbophos), monocrotophos, NIT, nitenpyram, *p*-nitrophenyl phosphate, matrine, methamidophos, paraoxon, paraoxon ethyl, parathion, parathion methyl, penfluoron, permethrin, phosalone, pirimiphos, procymidone, profenofos, pyridaben, pyrimicarb, quinalphos, teflubenzuron, triazophos, thiamethoxam, tribenuron methyl, trichlorfon, *o*,*p*′- and *p*,*p*′-1,1,1-trichloro-2,2-bis(*p*-chlorophenyl)ethylene (DDT), 1,1,1-trichloro-2,2-bis(4chlorophenyl)ethane, triflumuron, and thiacloprid; (iv) **plant growth hormones**: forchlorfenuron, 6-benzylaminopurin, indole-3-acetic acid, 3-indolebutyric acid, and indolepropionic acid.

In 2010, Wen et al. reported one of the first works about the application of MOFs in the efficient detection of agrochemicals. In this study, a new MOF, named [Cd(2,2′,4,4′-bptcH_2_)]_*n*_ (2,2′,4,4′-bptcH_4_: 2,2′,4,4′-biphenyltetracarboxylic acid), that was thermally stable and luminescent was prepared via a hydrothermal reaction.^122^ This material was tested as a solid-phase extraction (SPE) material for the detection of trace levels of organophosphate pesticide (OP) via stripping voltametric analysis. The determination of parathion methyl as a model included two main steps: parathion methyl adsorption and electrochemical stripping detection of adsorbed pesticide. The MOF modified glass carbon electrode was immersed into a sample solution containing the desired parathion methyl concentration, and the peak currents increased rapidly with the immersion time, up to 12 min, which indicated the saturation. The calculated limit of detection (LOD: 0.0006 μg·mL^–1^) is comparable with that of 0.0048 μg·mL^–1^ at a hanging mercury drop electrode, suggesting that the reported MOF is reliable for the determination of OPs in water. The same year, Barreto et al. reported the evaluation a new adsorbent 3D MOF [(La_0.9_Eu_0.1_)_2_(DPA)_3_(H_2_O)_3_] (H_2_DPA: pyridine-2,6-dicarboxylic acid) for the determination of pesticides from four chemical classes, namely, organochlorine (endosulfan), organophosphate (malathion and parathion methyl), dicarboximide (procymidone), and carbamate (pirimicarb) in fresh lettuce (*Lactuca sativa*) by matrix solid-phase dispersion (MSPD) and gas chromatography–mass spectrometry (GC/MS).^123^ The recoveries obtained ranged from 78% to 107% with relative standard deviation (RSD) values between 1.6% and 8.0%. The LOD and limit of quantification (LOQ) ranged from 0.02 to 0.05 mg·kg^–1^ and from 0.05 to 0.1 mg·kg^–1^, respectively, for the different pesticides studied. Importantly, the comparison with a conventional sorbent (silica gel) showed better performance of the MOF sorbent for all tested pesticides. However, the reasons of this improvement are not investigated or discussed by authors. Later on, in 2017, Tao et al.^124^ originally synthesized a tetraphenylethene-based ligand (BPyTPE: (*E*)-1,2-diphenyl-1,2-bis(4-(pyridin-4-yl)phenyl)ethene) with a *trans* conformation and prominent AIE properties. On the basis of BPyTPE, a novel 2D pillared-layered LMOF [Zn_2_(bpdc)_2_(BPyTPE)] (H_2_bpdc: biphenyl-4,40-dicarboxylic acid) was developed showing a 3-fold interpenetration structure. The activated material (without solve) exhibits a strong blue-green emission at 498 nm with an important φ_F_ of 99%. The emission of the MOF without solvent can be quenched selectively and effectively by 2,6-DN. Thus, the authors established a method to quantitatively and sensitivity detect trace 2,6-DN with a linear range of 0.94–16.92 ppm and a low detection limit of 0.13 ppm.

**Figure 7:**
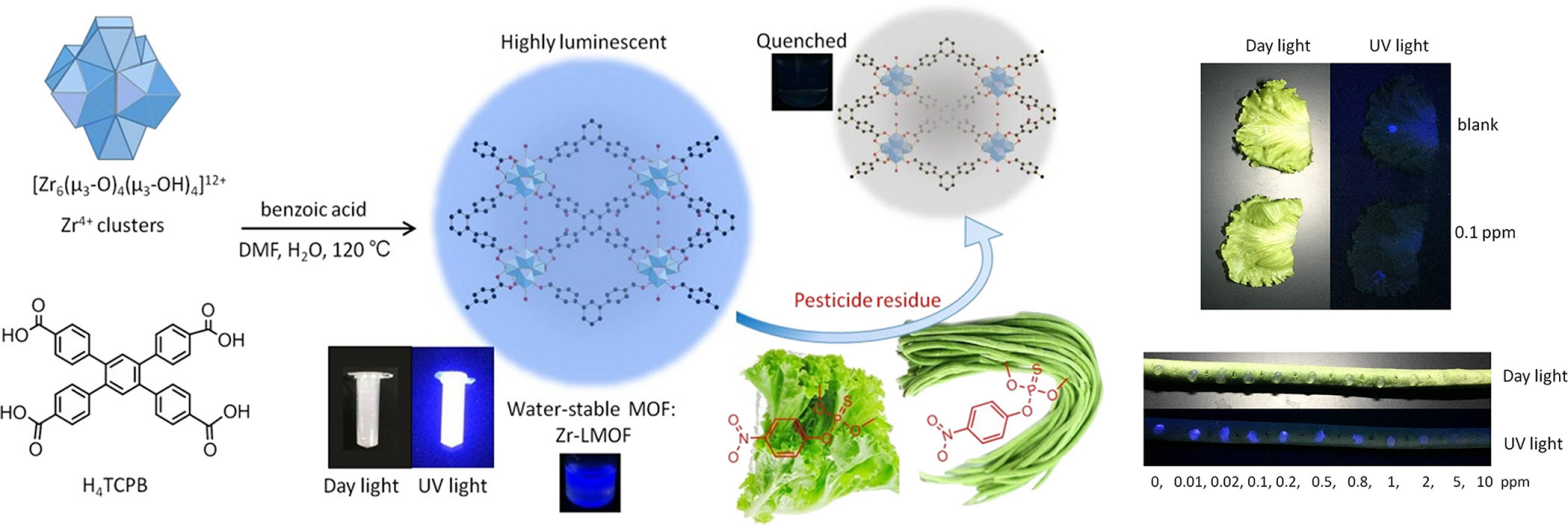
Schematic diagram of the synthesis of CAU-24 and its application for OP pesticide sensing. Insets show the blue fluorescence of the aqueous solution of this Zr-MOF before and after quenching by target parathion methyl in an aqueous solution and directly applied on the surface of vegetable surfaces. Reprinted with permission from ref (125). Copyright 2014 Elsevier B.V.

When one considers these outstanding original works, MOFs have opened a new opportunity for the development of efficient techniques to detect agrochemicals. However, most of these materials are more or less sensitive to moisture or water and can be degraded through hydrolysis. Only few MOFs can maintain their stability in water or a moist environment. One example is the previously reported luminescent Zr-MOF CAU-24, based on the C-centered orthorhombic arrangement cluster [Zr_6_(μ_3_-O)_4_(μ_3_-OH)_4_^12+^] bridged by TCPB^4–^ linkers in a scu topology (H_4_TCPB: 1,2,4,5-tetrakis(4-carboxyphenyl)benzene; *S*_BET_: 1450 m^2^·g^–1^; rhombic channels of ∼10 × 5.3 and ∼3.5 × 2.4 Å^2^, Figure 7). This material demonstrated a rapid, sensitive, and *in situ* detection of OP pesticides.^125^ Along the 22 pesticides tested, the synthesized CAU-24 quickly absorbs trace amounts of OP parathion methyl and indicates its presence. It has a low LOD of 0.115 μg·kg^–1^ (0.438 nM) with a wide linear range from 70 μg·kg^–1^ to 5.0 mg·kg^–1^. The water stability of this Zr-MOF was investigated by suspending it in water for 24 h and monitoring by powder X-ray diffraction (PXRD), adsorption/desorption isotherms, and pore distribution. The crystalline structure and porosity of the Zr-MOF was kept in water after 24 h. Finally, the Zr-MOF was used to mimic rapid *in situ* imaging detection of pesticide residues on surface vegetables (lettuce and cowpea); visual signals appeared under UV light within 5 min. Therefore, this MOF has the possibility for low-cost, rapid, and *in situ* imaging detection of OP contamination via easy-to-read visual signals.

**Figure 8:**
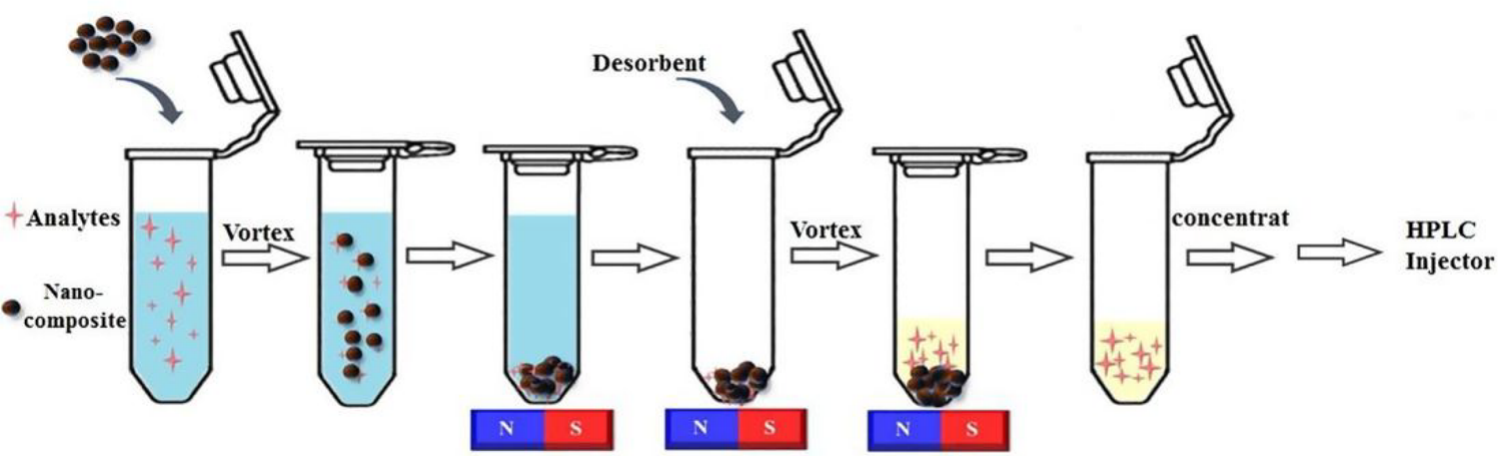
Schematic view of the MSPE procedure when using ATP@Fe_3_O4@@ZIF-8 in benzoylureas determination (N: North; S: South). Reprinted with permission from ref (127). Copyright 2020 Elsevier Ltd.

MOF composites have also been used in the determination of agrochemicals. In 2014, a MOF with an iron oxide enclosure was reported for the determination of OPs in biological samples.^126^ In this work, MIL-101(Fe) was modified as a model with superparamagnetic qualities using Fe_3_O_4_ to form a homogeneous magnetic product (Fe_3_O_4_/MIL-101 composite). The Fe_3_O_4_/MIL-101 composite was investigated for the magnetic solid-phase extraction of six OPs from human hair and urine samples followed by gas chromatography analysis. Under optimized conditions (desorption solvent, extraction time, desorption time, etc.), this method showed low LOD (0.21–228 ng·mL^–1^), wide linearity, and good precision (1.8–8.7% for intraday, 2.9–9.4% for interday). The adequate recoveries of the spiked samples were 76.8–94.5% and 74.9–92.1% for hair and urine, respectively, suggesting that the Fe_3_O_4_/MIL-101 sorbent is feasible for the analysis of trace OPs in biological samples. Other Fe_3_O_4_-MOF-based composites have recently been reported for the efficient determination of different agrochemicals, for example, the magnetic solid-phase extraction method based on the attapulgite-modified MOF (ATP@Fe_3_O_4_@ZIF-8) in the determination of bezoylureas (insecticides; see Table 3).^127^ ATP, an eco-friendly nature and low-cost clay, is added here to improve the hydrolytic stability of ZIF-8 as the −OH groups of ATP can selectively coordinate with the metal ions in ZIF-8. The ATP@Fe_3_O_4_@ZIF-8 nanocomposite was applied as a sorbent for the magnetic solid-phase extraction (MSPE) of benzoylureas prior to high-performance liquid chromatography (HPLC) determination (Figure 8). The established method was validated in terms of linearity (2.5–500 μg L^–1^ with satisfactory recovery of 88.29–95.99%) and precision (relative standard deviation, RSD, of <8%). Moreover, after 5 cycles, there was hardly any noticeable loss of the extraction efficiency. Finally, this method was effectively used in the determination of 6 benzoylureas in different tea infusions; the determined relative recoveries ranged from 78.8% to 114.3%.

**Table 3:** Reported MOFs and MOF Composites Related to the Detection of Agrochemicals^a^

agrochemical	MOF/MOF composite	recovery (%)	applicability	detection limit	ref, year
aldrin	MOF-199/GO fiber	90.6–104.4	river water	2.3–6.9 × 10^–3^ ppm	(130), 2013
chlordane		82.7–96.8	soil		
*p*,*p*′-DDE		72.2–107.7	water convolvulus		
*p*,*p*′-DDD		82.8–94.3	longan		
dieldrin					
endosulfan					
heptachlor epoxide					
hexachlorobenzene					
aldrin	needle trap device packed with the MIL-100(Fe)		air environment	0.04–0.41 μg·m^–3^	(131), 2021
chlordane					
dieldrin					
*o*,*p*′-DDT					
*p*,*p*′-DDT					
hexachlorobenzene					
1,1,1-trichloro-2,2-bis(4chlorophenyl)ethane					
ametryn	MIL-101(Cr)	73.37–107.7 ± 0.10–14.58	corn	0.01–0.12 ng·g^–1^	(132), 2018
atraton					
desmetryn					
dipropetryn					
prometon					
prometryn					
ametryn	MIL-101(Cr)	91.1–106.7	soybean	1.56–2.00 μg·kg^–1^	(133), 2015
atraton					
atz					
chlorotoluron					
fenuron					
monuron					
terbuthylazine					
ametryn	MIL-101(Cr)	89.5–102.7	peanuts	0.98–1.9 μg·kg^–1^	(134), 2014
atraton					
ATZ					
chlortoluron					
monuron					
terbumeton					
terbuthylazine					
ametryn	Fe_3_O_4_@MIL-100(Fe)	97.6–101.5	environmental water and vegetable samples	2.0–5.3 ppb	(135), 2018
ATZ					
prometon					
simazine					
amidosulfuron	UiO-66-NH_2_	75.7–94.2	spiked soil	0.19–1.79 ppb	(136), 2017
metsulfuron methyl		82.2–95.3	water		
sulfosulfuron					
thifensulfuron methyl					
amidosulfuron	UiO-66-NH_2_ magnetic stir bar	68.8–98.1	water and soil	0.04–0.84 ppb	(137), 2018
metsulfuron methyl					
sulfosulfuron					
tribenuron methyl					
thifensulfuron methyl					
ATZ	magG@PDA@Zr-MOF	29–95	tobacco	10.78–45.45 ng·g^–1^	(138), 2018
bifenthrin					
cyhalothrin					
parathion methyl					
penconazole					
pirimiphos					
procymidone					
trifluralin					
atraton	Fe_3_O_4_@SiO_2_-GO/MIL-101(Cr)	83.9–103.5	rice	0.010–0.080 μg kg^–1^	(139), 2018
ATZ					
prometon					
secbumeton, terbuthylazine					
terbumeton					
trietazine					
ATZ	ZIF-8/SiO_2_@Fe_3_O_4_	88.0–101.9	fruit, vegetables, and water	0.18–0.72 ppb	(140), 2017
prometryn					
ATZ	[Mg_2_(APDA)_2_(H_2_O)_3_]		DMF solutions	150 ppb	(141), 2018
carbaryl					
chlorpyrifos					
2,4-dichlorophenol					
2,6-DN					
ATZ	[(La_0.9_Sm_0.1_)_2_(DPA)_3_(H_2_O)_3_]	52.7–135.0	peppers (*Capsicum annuum L*.)	16.0–67.0 μg·kg^–1^	(142), 2018
bifenthrin					
bromuconazole					
clofentezine					
fenbuconazole					
flumetralin					
pirimicarb					
procymidone					
avermectin	Zn-BTC	78.6–116.1 for industrial wastewater	wastewater	0.20–1.60 ppb	(143), 2017
carbofuran		87.5–107.9 for domestic sewage			
clorpirifos		97.5–101.1 for tap water			
fenvalerate					
pyridaben					
triadimefon					
azinphos methyl	[Y_1.8_Eu_0.1_Tb_0.1_(1,4-PDA)_3_(H_2_O)_1_]		aqueous media	212 ppb	(144), 2019
azinphos-methyl	[Cd_2.5_(1,4-PDA)(tz)_3_]		aqueous media	16 ppb	(145), 2017
azinphos-methyl	[Cd_3_(1,4-PDA)_1_(tz)_3_Cl(H_2_O)_4_]		apple and tomato	8 ppb	(146), 2018
chlorpyrifos					
parathion					
bensulfuron methyl	MIL-53-PVDF MMM	77.20–111.00	tap, surface, and seawater	3.75–10.30 × 10^–3^ ppm	(147), 2019
chlorimuron ethyl nicosulfuron					
metsulfuron methyl					
pyrazosulfuron ethyl					
thifensulfuron methyl					
bensulfuron methyl	MIL-101(Fe)@PDA@Fe_3_O_4_	87.1–108.9	real water samples (lake, river, irrigation, and reservoir water) and vegetables (pak choi, spinach, and celery)	0.12–0.34 ppb	(148), 2018
chlorimuron ethyl					
pyrazosulfuron ethyl					
sulfometuron methyl					
6-benzylaminopurin	ZIF-8@SiO_2_	70–120	oranges	3.0–59.4 ppb	(149), 2018
indole-3-acetic acid					
indolepropionic acid					
3-indolebutyric acid					
bifenthrin	UiO-66	60.9–117.5	vegetables	0.4–2.0 ng·g^–1^	(150), 2021
fenvalerate					
isocarbophos					
parathion					
permethrin					
triazophos					
bifenthrin	MIL-101(Cr)-based composite	78.3–103.6	environmental water and tea samples	0.008–0.015 ppb	(151), 2018
deltamethrin					
fenpropathrin					
permethrin					
bifenthrin	[(Nd_0.9_Eu_0.1_)_2_(DPA)_3_(H_2_O)_3_]	78–88	soursop exotic fruit (*Annona muricata*)	0.03–0.05 mg·kg^–1^	(152), 2015
teflubenzuron					
thiacloprid					
thiamethoxam, thiophanate methyl					
bromopropylate	[Zn(BDC)_*x*_(NH_2_-BDC)_1–*x*_(H_2_O)_2_]*n*	47–76	coconut palm	0.01–0.05 μg·g^–1^	(153), 2017
clofentezine					
coumaphos					
difenoxuron					
diniconazole					
flumetralin					
fluometuron					
teflubenzuron					
butachlor	MIL-101(Zn)	86.9–119.0	black, red, and kidney beans	1.18 μg·kg^–1^	(154), 2019
metazachlor				0.58 μg·kg^–1^	
pretilachlor				1.78 μg·kg^–1^	
propanil				0.90 μg·kg^–1^	
butralin	Fe_3_O_4_@NH_2_-MIL-101	70.5–119.8	aqueous solutions	0.13–0.86 ppb	(155), 2020
chlorothalonil					
chlorpyrifos					
deltamethrin					
pyridaben					
tebuconazole					
carbendazim	MXene/CNHs/β-CD-MOFs	97.77–102.01	aqueous solution with coexisting substrates and tomato	1.0 nM	(129), 2020
carbendazim	UiO-67	90.82–103.45	apple, cucumber, and cabbage	3.0 × 10^–3^ μM	(156), 2021
carbaryl	MIL-101(Fe)@GO	98.8–104.7	fruit and vegetables	1.2 and 0.5 nM	(157), 2019
carbofuran					
carbaryl	F1, F2, F3, and F4		aqueous solution	–, 108, 106, and 30 ppb	(158), 2021
matrine					
triadimefon					
chloramphenicol	MIP/Zr-LMOF	95–105	milk and honey	13 ppb	(159), 2021
chlorfluazuron	ATP@Fe_3_O_4_@ZIF-8	78.8–114.3	tea infusions	0.7–3.2 ppb	(127), 2020
flufenoxuron					
hexaflumuron					
lufenuron					
teflubenzuron					
triflumuron					
chlorothalonil	(H_3_O)[Zn_2_L_1_(H_2_O)]		aqueous solutions	2.93 ppm	(160), 2019
2,6-DN					
nitrofen					
trifluralin					
chlorpyrifos	AChE@Basolite Z1200		tomato	3 ng·L^–1^	(161), 2021
chlorpyrifos	Tb-MOF	82.17–93.6	tap water, cucumber, cabbage, kiwifruit, and apple	3.8 nM	(162), 2019
chlorpyrifos	[Ln(tftpa)_1.5_(2,2′-bpy) (H_2_O)]		ethanolic solutions, 5 cycles	0.14 ppb	(163), 2018
chlorpyrifos	UiO-66-NH_2_/Glycine/GO		aqueous solution	0.15 ppb	(164), 2021
chlorpyrifos	CBZ-BOD@ZIF-8		aqueous solution	1.15 ng·mL^–1^	(165), 2021
chlorpyrifos	TMU-4/PES	88–108	water and soil samples	5–8 ppb	(166), 2018
diazinon					
fenitrothion					
malathion					
chlorpyrifos	Cu/CuFe_2_O_4_@MIL-88A(Fe)	88.3–100.4	water samples (farm water, water of rice field, and river water) and fruit juice and vegetable samples (pomegranate, kiwi, orange, tomato, and cucumber)	0.2 and 0.5 ng mL^–1^	(167), 2021
phosalone					
chlorfluazuron	Fe_3_O_4_@MOF-808	84.6–98.3	tea beverages and juice samples	0.04–0.15 ppb	(168), 2020
clofentezine diflubenzuron					
forchlorfenuron					
hexaflumuron					
lufenuron					
penfluoron					
clethodim	MIL-125(Ti)-NH_2_@TiO_2_	96.8–103.5	aqueous solutions	10 nM	(169), 2015
cyhalothrin	MOFs-MIPs-MSPD	>93	wheat	1.8–2.8 ng g^–1^	(170), 2019
β-cyfluthrin					
cyphenothrin					
2,4-D	MOF-808	77.1–109.3	mixed juice, orange juice, and tap water	0.1–0.5 ppb	(171), 2019
2-DPP					
4-CPA					
dicamba					
2,4-D	UiO-66@cotton	83.3–106.8	cucumber and tap water	0.1–0.3 ppb	(172), 2020
2-DPP					
4-CPA					
dicamba					
2,4-D	UiO-67	86.12–103.44	tomato, cucumber, and white gourd	0.1–0.5 ppb	(173), 2018
2-DPP					
4-CPA					
dicamba					
2,4-D	UiO-66-NH_2_	82.3–102	tomato, Chinese cabbage, and rape	0.16–0.37 ng·g^–1^	(174), 2017
MCPA					
MCPB					
MCPP					
*p*,*p*′-DDD	M-M-ZIF-67	75.1–112.7	tap, river, and agricultural irrigation water samples	0.07–1.03 ppb	(175), 2018
*o*,*p*′- and *p*,*p*′-DDE					
*o*,*p*′- and *p*,*p*′-DDT					
α-, β-, γ-, and δ-HCH					
diazininon	UiO-66	85.7–97.8	tap and river water and tomato, apple, and tomato juice	2.5 ng·mL^–1^	(176), 2021
diazinon	MIL-101@GO-HF-SPME	88–104	tomato, cucumber, and agricultural water	0.21 ppm	(177), 2020
chlorpyrifos				0.27 ppm	
diazinon	ZIF-8	91.9–99.5	tap, waste, and river waters and apple, peach, and grape juices	0.03–0.21 ppb	(178), 2019
fenthion	Zn-based MOFs				
fenitrothion					
profenofos					
phosalone					
diniconazole	Fe_3_O_4_-MWCNT@MOF-199	62.80–94.20	eabbage, spinach, and orange and apple juices	520–1830 ppb	(179), 2021
fenbuconazole					
flusilazole					
hexaconazole					
penconazole					
propiconazole					
tebuconazole					
2,6-DN	[Zn_2_(L)_2_(TPA)]		recyclable (5 cycles), detection in methanol or chloroform solutions	0.39 ppm	(180), 2019
2,6-DN	[Zn_2_(bpdc)_2_(BPyTPE)]		dichloromethane	0.13–0.8 ppm	(124), 2017
2,6-DN	[Cd(tptc)_0.5_(bpz)(H_2_O)]		aqueous media	638 ppb	(181), 2020
2,6-DN	Cd-CBCD		aqueous media; recyclability (5 cycles)	145 ppb	(182), 2019
2,6-DN	[Ag(CIP^–^)]		DMF	1.7 × 10^–7^ M	(183), 2019
2,6-DN	[Ln_3_(HDDB)(DDB)(H_2_O)_6_]	98–103.1	aqueous solution, nectarines, carrots, and grapes	86 ppb	(184), 2021
	(Ln = Eu, Tb, Dy, Gd)				
2,6-DN	[Eu_2_(dtztp)(OH)_2_(DMF)(H_2_O)_2.5_]		lake water, 5 cycles	5.28 ppm	(185), 2021
dichlorvos	Fe_3_O_4_/MIL-101	76.8–94.5	hair	0.21–2.28 ppb	(126), 2014
methamidophos		74.9–92.1	urine		
dimethoate					
malathion					
parathion					
parathion methyl					
difenoconazole	M-IRMOF	74.82–99.52	vegetable	0.25 ppb	(186), 2019
epoxiconazole				0.25 ppb	
fenbuconazole				1.0 ppb	
pyraclostrobin thiabendazole				0.25 ppb	
				0.25 ppb	
diniconazole	UiO-66@polymer	90.4–97.5	water	1.34–14.8 × 10^–3^ ppm	(187), 2019
flutriafol		84.0–95.3	soil		
hexaconazole					
pyrimethanil					
tebuconazole					
diniconazole	MOF-5@GO	85.6–105.8	grape, apple, cucumber, celery, cabbage, and tomato	0.05–1.58 ng·g^–1^	(188), 2016
hexaconazole					
myclobutanil					
propiconazole					
triadimefon					
diniconazole	defective UiO-66	82.6–92.2, 82.8–98.2, and 80.2–88.2 for pond, river, and lotus pond waters	environmental water samples	4–36 ppb	(189), 2021
pyrimethanil					
tebuconazole					
dinotefuran	[(CH_3_)_2_NH_2_]_2_[Cd_3_(BCP)_2_]		water	2.09 ppm	(190), 2021
α- and β-endosulfan	[(La_0.9_Eu_0.1_)_2_(DPA)_3_(H_2_O)_3_]	70–107	lettuce	0.02 mg·kg^–1^	(123), 2010
malathion					
parathion methyl					
procymidone					
pyrimicarb					
epoxiconazole	Fe_3_O_4_@APTES-GO/ZIF-8	71.2–110.9	tap water, honey samples, and mango, grape, and orange juices; recyclability (5 cycles)	0.014–0.109 ppb	(128), 2020
flusilazole					
tebuconazole					
triadimefon					
fenitrothion	[Cd(BDC-NH_2_)(H_2_O)_2_]_*n*_		ethanolic solutions	1 ppb	(191), 2014
parathion methyl					
paraoxon					
parathion					
fenitrothion	MOF-5		aqueous solutions	5 ppb	(192), 2014
parathion methyl					
paraoxon					
parathion					
fenitrothion	SPP@Au@MOF-5	97.5	soil	10^–12^ M	(193), 2019
paraoxon ethyl					
GLY	MOF-Calix		aqueous solutions	0.38 ppm	(194), 2020
GLY	[Tb(L)_2_NO_3_]_*n*_ (HL)		aqueous solutions	0.0144 μM	(195), 2021
glufosinate	MOF-545		aqueous solutions	0.0009 ppb	(196), 2021
GLY					
imidacloprid	UiO-66-NH_2_	92.39	fruit samples	40–60 ppb	(197), 2021
thiamethoxam		94.37			
iprodione	MIL-101-NH_2_@Fe_3_O_4_-COOH	71.1–99.1	real water samples	0.04–0.4 ppb	(198), 2018
myclobutanil					
prochloraz					
tebuconazole					
malathion	BTCA-P-Cu-CP	91.0–104.4	vegetable extracts (spinach, celery, lettuce, red capsicum, eggplant, and cherry tomato)	0.17–0.59 nM	(199), 2019
malathion	Basolite C300	>92%	water, fruits, and vegetables	4.0 ppb	(200), 2020
malathion	Pt@UiO-66-NH_2_	93.34–97.80	aqueous solutions	4.9 × 10^–15^ M	(201), 2019
MCPA	HKUST-1	57–100	water, soil, rice, and tomato	10 × 10^–3^ ppm	(202), 2018
monocrotophos, trichlorfon	MIL-101(Cr)@MIP	86.5–91.7	apple and pear	0.011 mg·kg^–1^	(203), 2017
				0.015 mg·kg^–1^	
molinate	ZIF-67@MgAl_2_O_4_		aqueous solutions	3 ppm	(204), 2021
nicosulfuron	Tb-BDOA		aqueous solutions	1.61	(205), 2021
thiamethoxam				1.04 μM	
nitrofen	PVP/Glu/CRL@ZIF-8	92.15–107.58	aqueous solutions	0,14 μM	(206), 2021
*p*-nitrophenyl phosphate	[Co(OBA)(2,2′-BPY)]	93.6–131.6	fruits (watermelon, orange, tomato, and apple)	352 nM (0.07 mg kg^–1^)	(207), 2021
bis(*p*-nitrophenyl) phosphate			real water samples		
parathion methyl	[Cd(2,2′,4,4′-bptcH_2_)]*_n_*		aqueous solutions	0.006 ppb	(122), 2010
parathion methyl	ZnPO-MOFs	93.0–104.6	irrigation water	0.12 μg kg^–1^ (0.456 nM)	(208), 2018
parathion methyl	Au/Cys-Fe_3_O_4_/MIL-101		juice samples	5 ppb	(209), 2021
parathion methyl	Zr-BDC-rGO	95.3–103.4	aqueous solutions	0.5 ng mL^–1^	(210), 2021
parathion methyl	Ru(bpy)_3_^2+^-ZIF-90	93.3–103.6	aqueous solutions	0.037 ng mL^–1^	(211), 2021
paraquat	[Zn_2_(cptpy)(BTC)(H_2_O)]*_n_*		aqueous solutions	9.73 × 10^–6^ M	(212), 2019
parathion methyl	Zr-LMOF	78–107	cowpea and lettuce	0.115 μg·kg^–1^	(125), 2019
parathion					
parathion	[Cd(BDC-NH_2_)(H_2_O)_2_]_*n*_		rice	0.1 ppb	(213), 2015
quinalphos	CD@UiO-66-NH_2_	98–105	tomato and rice	0.3 nM	(214), 2021
NIT	PCN-224	97.76–104.02	paddy water	0.03 × 10^–3^ ppb	(62), 2020
		88.1–100.30	soil		
NIT	Rho B@1	95.2–102.0	river water	0.27 μg·kg^–1^	(215), 2020
	Rho 6G@1	93.4–103.5		0.86 μg·kg^–1^	
thiabendazole	Tb^3+^@UiO-66-(COOH)_2_	98.41–104.48	orange and aqueous solutions	0.271 μM	(216), 2021
thiabendazole	Ag-Au-IP6-MIL-101(Fe)	84.4–112.8	juice	50 ppb	(217), 2019

a The table is organized according to the agrochemical studied, followed by the MOF-based material name (or chemical formula), recovery (%), applicability, and detection limit. 2,2′-BPY: 2,2′-bipyridyl; 2,2′,4,4′-bptcH_2_: 2,2′,4,4′-biphenyltetracarboxylic acid; AChE: acetylcholinesterase; APTES: (3-aminopropyl)triethoxysilane; ATP: attapulgite; BPyTPE: (*E*)-1,2-diphenyl-1,2-bis(4-(pyridin-4-yl)phenyl)ethene; bpz: 2-(1*H*-pyrazol-3-yl)pyridine; CD: carbon dots; CNHs: carbon nanohorns; CP: coordination polymer; CRL: *Candida rugosa lipase*; DMF: *N*,*N*′-dimethylformamide; GO: graphene oxide; H_2_BDC: bezene-1,4-dicarboxylic acid; H_2_BDC-NH_2_: 2-aminoterephthalic acid; H_2_bpdc: biphenyl-4,4′-dicarboxylic acid; H_2_DPA: pyridine-2,6-dicarboxylic acid; H_3_BTC: 1,3,5-benzenetricarboxylic acid; H_4_BTCA: benzene-1,2,4,5 tetracarboxylic acid; HCIP: 4-(4-carboxylphenyl)-2,6-di(4-imidazol-1-yl)phenyl pyridine; H_3_CBCD: 4,4′-(9-(4′-carboxy-[1,1′-biphenyl]-4-yl)-9*H*-carbazole-3,6-diyl)dibenzoic acid; H_4_dtztp: 2,5-bis(2*H*-tetrazol-5-yl) terephthalic acid; Hcptpy: 4-(4-carboxyphenyl)-2,2′:4′,4″-terpyridine; H_5_DDB: 3,5-di(2′,4′-dicarboxylphenyl) benzoic acid; HL: 3.5-bis(triazol-1-yl) benzoic acid; H_2_tftpa: tetrafluoroterephthalic acid; H_4_tptc: *p*-terphenyl-2,2′,5″,5‴-tetracarboxylate acid; H_2_APDA: 4,4′-(4-aminopyridine-3,5-diyl)dibenzoic acid; H_4_BCP: 5-(2,6-bis(4-carboxyphenyl)pyridin-4-yl)-isophthalic acid; HF: hollow fiber; IP_6_: inositol hexaphosphate; L_1_H_5_: 2,5-(6-(4-carboxyphenylamino)-1,3,5-triazine-2,4-diyldiimino)diterephthalic acid; L: 4-(tetrazol-5-yl)phenyl-4,2′:6′,4″-terpyridine; polymer: poly(*N*-vinylcarbazole-*co*-divinylbenzene); magG: magnetic graphene; MIP: molecularly imprinted polymer; MMM: mixed-matrix membranes; MSPD: matrix solid-phase dispersion; OBA: 4,4′-oxybis(benzoic acid); PBS: phosphate buffer saline; PDA: polydopamine; 1,4-PDA: 1,4-phenylenediacetate; PES: poly(ether sulfone); PVDF: poly(vinylidene fluoride); Rho: rhodamine; SPME: solid-phase microextraction; SPP: surface plasmon polariton; TPA: terephthalic acid; tz: 1,2,4-triazolate.

Another example of magnetic solid-phase extraction using Fe_3_O_4_@ZIF-8-based composites is the work reported by Senosy et al. on the basis of the synthesis of Fe_3_O_4_@APTES-GO/ZIF-8 (APTES: (3-aminopropyl)triethoxysilane; GO: graphene oxide) and its evaluation as an adsorbent for the determination of triazole fungicides in water, honey, and fruit juices.^128^ Here, GO sheets were used to improve the dispersion of the adsorbent in aqueous solutions and, again, ZIF-8 to ensure enough surface area and active sites. Under the optimum conditions (extraction time, pH value of the sample, etc.), the obtained linearity of this method ranged from 1 to 1000 μg·L^–1^ for all analytes. The LODs and LOQs of four triazole fungicides ranged from 0.014 to 0.109 μg L^–1^ and from 0.047 to 0.365 μg L^–1^, respectively. Moreover, this adsorbent could be reused without significant loss of its extraction recoveries. When compared with the outcomes from other studies, Fe_3_O_4_@APTES-GO/ZIF-8-MSPE could provide a higher performance and achieve satisfactory results for the analysis of trace triazole fungicides in complex matrices. Another composite, based on a nanoarchitecture of Mxene/carbon nanohorns/β-cyclodextrin-MOF (MXene/CNHs/β-CD-MOFs), was utilized as an electrochemical sensing platform for the determination of carbendazim pesticide.^129^ β-CD-MOFs combined the properties of the host–guest recognition of β-CD and porous structure, high porosity, and pore volume of MOFs, which are fundamental in achieving a high adsorption capacity of carbendazim. MXene/CNHs possess a large specific surface area, accessible active sites, and high conductivity, which allowed more mass transport channels and enhanced the mass transfer capacity and catalysis of carbendazim.^123,126,128^ With the collaborative effect of both (β-CD-MOFs and MXene/CNHs), the electrode extended a wide linear range from 3.0 nM to 10.0 μM and a low LOD of 1.0 nM. Additionally, this sensor also showed high selectivity, reproducibility, and long-term stability as well as satisfactory application in tomato samples.

## 7 Perspectives in Using MOFs in Agriculture

As a novel class of materials, MOFs exhibit a great potential in agroindustry, either to detect or eliminate agrochemicals or to achieve their sustained and controlled release. In all these scenarios, the aim is to reach the rational and environmentally friendly use of agrochemicals. Despite the novelty of MOFs in agriculture, the experience acquired in other areas (particularly biomedical and environmental ones) allow us to identify precise challenges related to their use in agriculture.

First, MOF stability under the working conditions is of crucial relevance. However, from the wide number of MOFs and MOF-based composites reported in environmental remediation (water and soil), only few discuss this critical point and mostly under conditions far from real water streams or fields. In this sense, many of these materials are built up from toxic metals (e.g., Cr, Ag) and/or harmful organic moieties (e.g., porphyrins), which can be released upon the MOF degradation. The selection of safe and stable MOFs is therefore mandatory for their use in agroindustry (mainly for environmental remediation and agrochemical controlled release). Further, it is essential to investigate the performance of MOFs under real conditions using complex water and soil compositions and/or vegetables or plants (e.g., river water, real fields or greenhouses, vegetables, products, etc.), considering concentration ranges found in nature, different temperatures, humidity, sunlight hours, soil composition, or pH in different parts of plants, among others.

Second, the cost of MOFs is of particular importance for agroindustry applications. When one takes into account that vegetables and fruits are normally popular and affordable, it is necessary to use a low-cost and long-lifetime material. Nontoxic and abundant safe precursors together with simple synthetic routes with a high space time yield (STY; kilogram of MOF produced per cubic meter of reaction per day) (toxic solvents, expensive ligands, etc.) need to be put in place for the most promising candidates. Note here that few MOFs have been produced so far at the ton scale by different companies, and thus, they are not currently commercially available.^218^ To further progress through the application, specific manufacturing and devices should be considered (pellets, columns, membranes, etc.), and one needs to take into account the potential decrease in the MOF performance.

Finally, understanding the interaction of the agrochemicals and MOFs might help one further improve the resulting performances at the detection, removal, or progressive release stages. Also, research could be focused on multifunctional MOFs and MOF composites that combine, for instance, the extraction with the detection of pesticides in food matrices or the simultaneous elimination of different agrochemicals.

Although there are challenges to the use of MOFs in agriculture, this new domain in the application of MOFs will continue, and it is expected that novel knowledge and development will soon be the outcome. This Review opens fascinating perspectives for the safe and efficient MOF application in agriculture.

## Abbreviations

2,2′-BPY: 2,2′-bipyridyl
1,3-DCPP: *cis*-1,3-dichloropropene
1,4-PDA: 1,4-phenylenediacetate
2-DPP: desmetryn,2-(2,4-dichlorophenoxy)propionic acid
2,2′,4,4′-bptcH_4_: 2,2′,4,4′-biphenyltetracarboxylic acid
2,4-D: 2,4-dichlorophenoxyacetic acid
2,6-DN: 2,6-dichloro-4-nitroaniline
4-CPA: 4-chlorophenoxyacetic acid
β-CD: β-cyclodextrin
AChE: acetylcholinesterase
AMPA: aminomethylphosphonic acid
APTES: (3-aminopropyl)triethoxysilane
ATP: attapulgite
ATZ: atrazine
Bp: black phosphorus
Bpd: 1,4-bis(4-pyridyl)-2,3-diaza-1,3-butadiene
BPyTPE: (*E*)-1,2-diphenyl-1,2-bis(4-(pyridin-4-yl)phenyl)ethene
bpz: 2-(1*H*-pyrazol-3-yl)pyridine
BSA: bovine serum albumin
C: chitosan
CA: cellulose acetate
CDs: carbon dots
CMCS: carboxymethyl chitosan
CNHs: carbon nanohorns
CP: coordination polymer
CPO: chloroperoxidase enzyme
CRL: *Candida rugosa lipase*
DDE: dichloro-2,2-bis(*p*-chlorophenyl)ethylene
DDT: 1,1,1-trichloro-2,2-bis(*p*-chlorophenyl)ethylene
DMF: *N*,*N*′-dimethylformamide
DUR: diuron
ED_50_: concentration for 50% of maximal effect
EDTA: ethylenediaminetetraacetate
EU: European Union
FAO: Food and Agriculture Organization
Fe-SPC: Fe-doped nanospongy porous biocarbon
GC/MS: gas chromatography–mass spectrometry
GLU: glufosinate
GLY: glyphosate
GO: graphene oxide
GSH: glutathione
H_2_APDA: 4,4′-(4-aminopyridine-3,5-diyl)dibenzoic acid
H_2_BDC: benzene-1,4-dicarboxylic acid
H_2_BDC-NH_2_: 2-aminoterephthalic acid
H_2_DPA: pyridine-2,6-dicarboxylic acid
H_2_bpdc: biphenyl-4,40-dicarboxylic acid
H_2_DPA: pyridine-2,6-dicarboxylic acid
H_2_TCPP: tetrakis(4-carboxyphenyl)porphyric
H_2_tftpa: tetrafluoroterephthalic acid
H_3_BTC: 1,3,5-benzenetricarboxylic acid
H_3_CBCD: 4,4′-(9-(4′-carboxy-[1,1′-biphenyl]-4-yl)-9*H*-carbazole-3,6-diyl)dibenzoic acid
H_4_BCP: 5-(2,6-bis(4-carboxyphenyl)pyridin-4-yl)-isophthalic acid
H_4_BTCA: benzene-1,2,4,5 tetracarboxylic acid
H_4_dtztp: 2,5-bis(2*H*-tetrazol-5-yl) terephthalic acid
H_4_TCPB: 1,2,4,5-tetrakis(4-carboxyphenyl)benzene
H_4_tptc: *p*-terphenyl-2,2″,5″,5‴-tetracarboxylate acid
H_5_DDB: 3,5-di(2′,4′-dicarboxylphenyl) benzoic acid
HCH: hexachlorocyclohexane
HCIP: 4-(4-carboxylphenyl)-2,6-di(4-imidazol-1-yl)phenyl pyridine
Hcptpy: 4-(4-carboxyphenyl)-2,2′:4′,4″-terpyridine
HF: hollow fiber
HKUST: Hong Kong University of Science and Technology
HL: 3.5-bis(triazol-1-yl) benzoic acid
HLF-1: human lung fibroblast
Hmim: 2-methylimidazole
HPLC: high-performance liquid chromatography
HRP: horseradish peroxidase enzyme
IC_50_: half maximal inhibitory concentration
ILCS: ionic liquid modified chitosan
Im: imidazonate
IP_6_: inositol hexaphosphate
IPU: isoproturon
KT_50_: knockdown time 50%
L_1_H_5_: 2,5-(6-(4-carboxyphenylamino)-1,3,5-triazine-2,4-diyldiimino)diterephthalic acid
L: 4-(tetrazol-5-yl)phenyl-4,2′:6′,4″-terpyridine
LC_50_: median lethal concentration
LOD: limit of detection
LOQ: limit of quantification
magG: magnetic graphene
MCPA: 2-methyl-4-chlorophenoxyacetic acid
MCPB: (2-methyl-4-chlorophenoxy) butyric acid
MCPP: (2-methyl-4-chlorophenoxy) propionic acid
MIP: molecularly imprinted polymer
M-M: magnetic multiwalled carbon nanotubes
MMM: mixed-matrix membranes
MOFs: metal–organic frameworks
MSPD: matrix solid-phase dispersion
MSPE: magnetic solid-phase extraction
NIR: near-infrared
NIT: nitenpyram
NND: neonicotinoids
NPs: nanoparticles
OBA: 4,4′-oxybis(benzoic acid)
OPs: organophosphorus pesticides
OPA: oxalate-phosphate-amine
P: pectin
PAN: polyacrylonitrine
PBS: phosphate buffer saline
PD: prochloraz (P) and 2,4-dinitrobenzaldehyde (D)
PDA: polydopamine
PES: poly(ether sulfone)
polymer: poly(*N*-vinylcarbazole-*co*-divinylbenzene)
*p*,*p*′-DDD: *p*,*p*′-1,1-dichloro-2,2-bis(*p*-chlorophenyl)ethylene
PS: photosensitizer
PVDF: poly(vinylidene fluoride)
XRD: powder X-ray diffraction
PyTBA: 4,4′,4′,4′-(pyrene-1,3,6,8-tetrayl)tetrabenzoate
QPE: quizalofop-P-ethyl
QpeH: quizolafop-P-ethyl hydrolase esterase
Rho: rhodamine
RSD: relative standard deviation
RT: room temperature
*S*_BET_: Brunauer–Emmett–Teller surface area
SL: sodium lignosulfonate
SPE: solid-phase extraction
SPME: solid-phase microextraction
SPP: surface plasmon polariton
STY: space time yield
TA: tannic acid
TCPB: 1,2,4,5-tetrakis(4-carboxyphenyl)benzene
Tebuc: tebuconazole
THI: thifluzamide
TMPyP: 5,10,15,20-tetrakis(1-methyl-4-pyridinio)porphyrin tetra(*p*-toluenesulfonate)
TPA: terephthalic acid
tz: 1,2,4-triazolate
us: ultrasound
*V*_p_: pore volume
ZIFs: zeolitic imidazolate frameworks
